# A Genetic Mosaic Screen Reveals Ecdysone-Responsive Genes Regulating *Drosophila* Oogenesis

**DOI:** 10.1534/g3.116.028951

**Published:** 2016-05-24

**Authors:** Elizabeth T. Ables, Grace H. Hwang, Danielle S. Finger, Taylor D. Hinnant, Daniela Drummond-Barbosa

**Affiliations:** * Department of Biochemistry and Molecular Biology, Bloomberg School of Public Health, Johns Hopkins University, Baltimore, Maryland 21205; † Division of Reproductive Biology, Bloomberg School of Public Health, Johns Hopkins University, Baltimore, Maryland 21205; ‡ Department of Biology, East Carolina University, Greenville, North Carolina 27858; § Department of Environmental Health Sciences, Bloomberg School of Public Health, Johns Hopkins University, Baltimore, Maryland 21205

**Keywords:** stem cells, germline, follicle cells, steroid hormone, nuclear hormone receptor

## Abstract

Multiple aspects of *Drosophila* oogenesis, including germline stem cell activity, germ cell differentiation, and follicle survival, are regulated by the steroid hormone ecdysone. While the transcriptional targets of ecdysone signaling during development have been studied extensively, targets in the ovary remain largely unknown. Early studies of salivary gland polytene chromosomes led to a model in which ecdysone stimulates a hierarchical transcriptional cascade, wherein a core group of ecdysone-sensitive transcription factors induce tissue-specific responses by activating secondary branches of transcriptional targets. More recently, genome-wide approaches have identified hundreds of putative ecdysone-responsive targets. Determining whether these putative targets represent *bona fide* targets *in vivo*, however, requires that they be tested via traditional mutant analysis in a cell-type specific fashion. To investigate the molecular mechanisms whereby ecdysone signaling regulates oogenesis, we used genetic mosaic analysis to screen putative ecdysone-responsive genes for novel roles in the control of the earliest steps of oogenesis. We identified a cohort of genes required for stem cell maintenance, stem and progenitor cell proliferation, and follicle encapsulation, growth, and survival. These genes encode transcription factors, chromatin modulators, and factors required for RNA transport, stability, and ribosome biogenesis, suggesting that ecdysone might control a wide range of molecular processes during oogenesis. Our results suggest that, although ecdysone target genes are known to have cell type-specific roles, many ecdysone response genes that control larval or pupal cell types at developmental transitions are used reiteratively in the adult ovary. These results provide novel insights into the molecular mechanisms by which ecdysone signaling controls oogenesis, laying new ground for future studies.

Steroid hormone signaling is critical for a wide variety of biological processes, including control of adult physiology and reproduction (Beato and Klug 2000; Pestka *et al.* 2013; Evans and Mangelsdorf 2014). In *Drosophila*, the steroid hormone ecdysone has been studied extensively for its biological roles and molecular function (Riddiford *et al.* 2000; King-Jones and Thummel 2005; Yamanaka *et al.* 2013; Belles and Piulachs 2014). Early experiments using larval salivary polytene chromosomes led to a hierarchical model of ecdysone signaling, wherein hormonal activation of the ecdysone receptor [a complex of the nuclear hormone receptors Ecdysone Receptor (EcR) and Ultraspiracle (Usp)] promotes the rapid expression of a small number of targets (Ashburner 1974). These so-called early-response genes encode transcription factors that activate a tissue-specific response to ecdysone by regulating a second set of targets (late-response genes). Among early-response genes, a core group of transcription factors, including *Ecdysone-induced protein 74EF* (*E74*), *Ecdysone-induced protein 75B* (*E75*), and *broad* (*br*), was identified via forward genetic screens, and subsequently demonstrated to modulate ecdysone signaling in a variety of cell types (King-Jones and Thummel 2005; Yamanaka *et al.* 2013). More recently, genome-wide approaches have been employed to identify putative ecdysone-responsive targets, and suggest that the transcriptional response to ecdysone is extremely diverse (Li and White 2003; Beckstead *et al.* 2005; Gauhar *et al.* 2009; Shlyueva *et al.* 2014b; Stoiber *et al.* 2016). The diversiform repertoire of target genes suggests that different cells are controlled by distinct subsets of ecdysone-responsive factors. Whether these putative targets represent *bona fide* targets *in vivo* must therefore be determined experimentally via traditional mutant analysis in a cell-type specific fashion.

The variety of well-described ovarian cell types, and the large range of cell biological processes controlling oogenesis make the *Drosophila* ovary an excellent model in which to directly compare the molecular mechanisms of ecdysone signaling across different cellular contexts. Ovaries are composed of 14–16 ovarioles, or strings of progressively more mature follicles each containing a developing oocyte (Figure 1A) (Spradling 1993). At the anterior end of each ovariole lies a germarium, which harbors two populations of adult stem cells that produce all of the cells in each follicle (Figure 1B). Germline stem cells (GSCs) divide asymmetrically to self-renew and produce a daughter cell, the cystoblast, which will undergo four additional rounds of mitotic division with incomplete cytokinesis to form a 16-cell cyst. One cell within the cyst is specified as the oocyte, while the other 15 differentiate as nurse cells. Somatic follicle stem cells (FSCs) also self-renew, and generate a variety of differentiated follicle cell types. Follicle cells encapsulate the developing 16-cell cyst in the posterior half of the germarium to individualize a new follicle.

**Figure 1:**
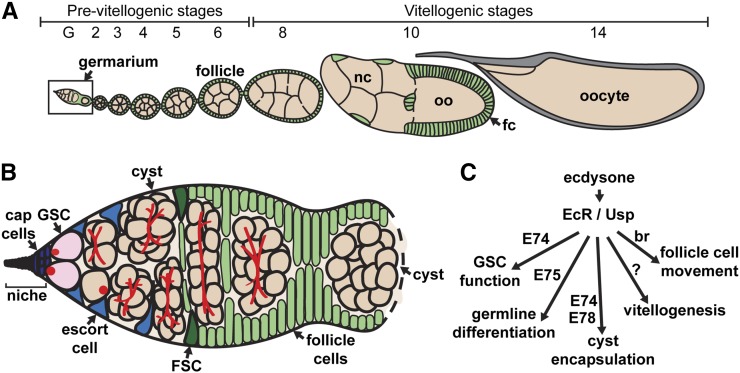
*Drosophila* oogenesis is fueled by the activity of germline stem cells. (A–B) The *Drosophila* ovary is composed of 14–16 ovarioles (A), each harboring a germarium (B) and older follicles that progress through 14 distinct stages of development. Within the germarium, germline stem cells (GSCs; pink) are juxtaposed to cap cells, the major cellular component of the somatic niche (purple), and a subset of escort cells (blue). GSCs divide to form daughter cells (cystoblasts), which divide four additional times to form 16-cell germline cysts (peach) composed of nurse cells (nc) and an oocyte (oo). Follicle stem cells (FSC; dark green) divide to form prefollicle cells, which surround the 16-cell germline cyst, and pinch away from the germarium, forming a follicle. Prefollicle cells give rise to a variety of specialized follicle cells (fc; green) that reside in an epithelial monolayer around each cyst. (C) Diagram of ecdysone pathway showing known ovarian responses to the hormone in *Drosophila*. Ecdysone-dependent events occurring during later stages of oogenesis, such as vitellogenesis and border cell migration, were not tested in this screen. See Introduction for additional details.

Ecdysone signaling has long been known to control the development of the *Drosophila* ovary, and to regulate multiple steps during adult oogenesis (Figure 1C) (Hodin and Riddiford 1998; Gancz *et al.* 2011; Belles and Piulachs 2014). Indeed, the major source of ecdysone in adult females is the ovary (Huang *et al.* 2008), and EcR and Usp are widely expressed throughout the germline and somatic lineages (Christianson *et al.* 1992; Buszczak *et al.* 1999; Carney and Bender 2000). Mutations affecting *EcR*, *usp*, and the early-response genes *E74*, *E75*, and *br* all result in impaired oogenesis (Belles and Piulachs 2014). For example, GSC proliferation and self-renewal intrinsically require ecdysone signaling, primarily through activation of *E74* (Ables and Drummond-Barbosa 2010). Germline differentiation, cyst formation, and cyst encapsulation also depend on ecdysone (Konig *et al.* 2011; Morris and Spradling 2012; Ables *et al.* 2015; Konig and Shcherbata 2015). Outside of the germarium, ecdysone signaling controls follicle growth and development, vitellogenesis, and the polarity, proliferation, migration, and survival of follicle cells (Buszczak *et al.* 1999; Tzolovsky *et al.* 1999; Bai *et al.* 2000; Carney and Bender 2000; Sun *et al.* 2008; Jang *et al.* 2009; Romani *et al.* 2009; Ables *et al.* 2015).

In this study, we compiled a list of ecdysone-responsive genes discovered in developing tissues, and performed a genetic mosaic screen to identify genes that control ovarian stem cell lineages. Our results demonstrate that, although ecdysone target genes are thought to be largely cell-type specific, genome-wide studies in distinct tissues can be used to identify candidate targets with roles in the ovarian germline and soma. Our studies also suggest that ecdysone response genes with roles in larval and pupal development may be used reiteratively in the ovary for similar biological processes, such as cell proliferation, cell movement, and the establishment and maintenance of cell identity. These results provide a foundation for future studies further investigating the molecular mechanisms of ecdysone signaling in the ovary.

## Materials and Methods

### Drosophila strains and culture

Flies were maintained at 22°–25° in standard medium (cornmeal/molasses/yeast/agar) supplemented with yeast. For genetic mosaic analyses using *flippase* (*FLP*)/*FLP recognition target* (*FRT*) (Xu and Rubin 1993), we obtained mutant alleles on *FRT*-containing chromosome arms from the BruinFly collection (Kyoto *Drosophila* Stock Center) (Call *et al.* 2007). For relative quantification of putative ecdysone response gene expression, we analyzed the temperature-sensitive *EcR^A483T^* in *trans* to null *EcR^M554fs^* (referred to as *EcR^ts^*) (Carney and Bender 2000) in parallel to heterozygous sibling controls following incubation at the restrictive temperature of 29° for 3 d. For RNAi experiments, *UAS-MESR3^GLC01393^* and *UAS-Tpr2^GLC01819^* (Ni *et al.* 2011) were crossed to *nos-Gal4* (*nos-GAL4*::*VP16-nos.UTR*) to reduce *MESR3* and *Tpr2* levels in the germline. Females carrying *nos-Gal4* alone were used as controls. Female progeny were collected 1–2 d after eclosion, and maintained for 5, 10, or 17 d at 25° on wet yeast paste prior to ovary dissection. Other genetic tools are described in FlyBase (Ashburner and Drysdale 1994).

### Genetic mosaic generation and stem cell analyses

Genetic mosaics were generated by *FLP/FRT*-mediated recombination in 2- to 3-d-old females carrying a mutant allele *in trans* to a wildtype allele (linked to a *Ubi-GFP* marker) on homologous *FRT* arms, and a *hs-FLP* transgene, as described (Ables and Drummond-Barbosa 2010; Laws and Drummond-Barbosa 2015). Briefly, flies were heat shocked at 37° two times per day for 3 d, and incubated at 25° for 12 d with daily transfers to freshly yeasted vials (standard media supplemented with dry yeast d 1–10, and wet yeast paste on the last 2 d prior to dissection). Wildtype alleles were used for generation of control mosaics. GSCs were identified based on the juxtaposition of their fusomes to the junction with adjacent cap cells (de Cuevas and Spradling 1998; Ables and Drummond-Barbosa 2010). FSCs were identified based on lineage tracing combined with morphology and position. Specifically, FSCs have a triangular nucleus and are the anterior-most cells within long-term follicle cell clones immediately anterior to the anterior-most lens-shaped cyst within each germarium (Nystul and Spradling 2007; Laws and Drummond-Barbosa 2015). Stem cell loss was measured as the percentage of total mosaic germaria showing evidence of recent stem cell loss, namely the presence of GFP-negative daughters (cystoblasts/cysts or follicle cells generated from an original GFP-negative stem cell) in the absence of the GFP-negative mother stem cell (Method I) (Ables and Drummond-Barbosa 2010; LaFever *et al.* 2010; Laws and Drummond-Barbosa 2015). Similar results were obtained by quantifying the frequency of total analyzed germaria containing at least one GFP-negative stem cell (Method II) (Xie and Spradling 1998; Laws and Drummond-Barbosa 2015). At least 50 germaria/ovarioles were scored for each mutant *FRT* line screened. Results were subjected to Chi-Square analysis using Microsoft Excel. Early germline cysts were identified based on fusome morphology (de Cuevas and Spradling 1998), and follicles were staged based on size and nuclear morphology as described (Spradling 1993). Additional phenotypes, including growth and encapsulation defects in the germline or soma, were noted in comparison with adjacent GFP-positive wildtype cells.

### Ovary immunostaining and microscopy

Ovaries were dissected, fixed, washed, and blocked as described (Ables and Drummond-Barbosa 2010). The following primary antibodies were used overnight at 4°: mouse anti-Hts [1B1, Developmental Studies Hybridoma Bank (DSHB); 1:10], mouse anti-Lamin C (LamC) (LC28.26, DSHB; 1:100), rabbit anti-GFP (TP401, Torrey Pines; 1:2500), and rat anti-Vasa (DSHB; 1:500). Following a 2-hr incubation with Alexa Fluor 488- or 568-conjugated goat species-specific secondary antibodies (Life Technologies; 1:200), ovaries were stained with 0.5 µg/ml 4′-6-diamidino-2-phenylindole (DAPI) (Sigma). Ovaries were mounted in 90% glycerol containing 20 mg/ml *n*-propyl gallate (Sigma). Confocal Z-stacks (1 µm optical sections) were collected with a Zeiss LSM700 microscope using Zeiss ZEN software. Images were analyzed, and minimally and equally enhanced via histogram using Zeiss ZEN software.

### RNA isolation and qRT-PCR

Whole ovaries were dissected in RNAlater (Ambion), and stored at –20° until RNA extraction. For each genotype, three RNA preparations (consisting of 10 pairs of ovaries each) were generated using the RNAqueous-4PCR Total RNA Isolation Kit (Ambion), and treated with TURBO DNase (Ambion) to remove genomic DNA, according to the manufacturer’s instructions. cDNA was synthesized from 500 ng of RNA using the iScript cDNA Synthesis kit (Bio-Rad), and used immediately for qPCR using primers listed in Supplemental Material, File S1. qPCR was conducted using iQ SYBR Green Supermix (Bio-Rad) on three technical replicates on a C1000 Touch Thermal Cycler equipped with a CFX96 Real-Time System (Bio-Rad). All primer efficiencies were between 100% and 120%. Normalized ΔΔCq values for each transcript were calculated against the reference gene *rp49* levels, and displayed relative to the indicated biological controls using CFX Manager (Bio-Rad).

### Data availability

The authors state that all data necessary for confirming the conclusions presented in the article are represented fully within the article.

## Results and Discussion

### Screen development and design

Genome-wide approaches to identify ecdysone-responsive genes in embryonic epidermal cells, ovarian somatic cells, and larval organs have yielded hundreds of putative targets (Li and White 2003; Beckstead *et al.* 2005; Gauhar *et al.* 2009; Shlyueva *et al.* 2014b; Stoiber *et al.* 2016). As steroid hormone signaling pathways can be cell-specific, it is unclear how many of these previously identified targets may function downstream of ecdysone in the various cell populations that compose the *Drosophila* ovary. Indeed, previous studies from our lab and others have demonstrated that, while ecdysone signaling controls many aspects of oogenesis (including GSC proliferation and maintenance, early germ cell differentiation, follicle encapsulation, and follicle survival), the requirement for early-response genes downstream of EcR activation for each of these functions varies (Figure 1C) (Hodin and Riddiford 1998; Buszczak *et al.* 1999; Carney and Bender 2000; Ables and Drummond-Barbosa 2010; Gancz *et al.* 2011; Konig *et al.* 2011; Morris and Spradling 2012; Ables *et al.* 2015).

**Figure 2:**
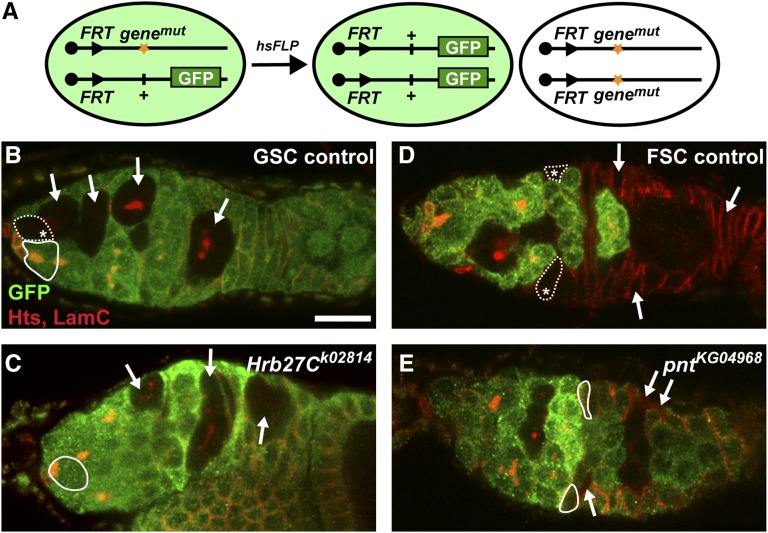
Putative ecdysone-responsive genes are required for GSC and FSC maintenance. (A) The *FLP/FRT* technique was used to generate genetic mosaics. Mitotic recombination is mediated by heat-shock-induced expression of *flippase* (*hsFLP*). Homozygous mutant (mut) cells are identified by the absence of a GFP marker, which is linked to the wildtype allele. (B–E) Representative control mosaic (B, D) or ecdysone-responsive mutant mosaic (C, E) germaria labeled with anti-GFP (green), anti-Hts (red; fusomes and follicle cell membranes), and anti-LamC (red; nuclear envelope of cap cells). Dotted lines and asterisks demarcate wildtype GFP-negative GSCs (B) or FSCs (D); solid lines demarcate GFP-positive GSCs (B–C) or FSCs (D–E). In control mosaic germaria, where all cells are genetically wildtype, GFP-negative daughter germ cells (arrows, B) and follicle cells (arrows, D) co-exist with GFP-negative GSCs and FSCs. In *Hrb27C* mutant mosaics (C), GFP-negative daughter germ cells are frequently observed in the absence of their GFP-negative mother GSC. Similarly, *pnt* mutant mosaics (E) are frequently observed with GFP-negative follicle cells, but without a GFP-negative mother FSC. Scale bar, 10 µm.

Many ecdysone-response genes described to date are essential for larval or pupal development; however, their potential roles in oogenesis remain largely unexplored. We therefore screened previously identified ecdysone targets (Beckstead *et al.* 2005; Gauhar *et al.* 2009) for ovarian roles using the *Flippase* (*FLP*)/*FLP Recognition Target* (*FRT*) genetic mosaic technique (Xu and Rubin 1993). This lineage-tracing system allows for the generation of clonal populations of homozygous mutant cells in otherwise heterozygous animals. The *FLP*/*FRT* system is particularly amenable to the dissection of gene function in the germline and follicle stem cell lineages in the ovary (Laws and Drummond-Barbosa 2015). For example, the *FLP*/*FRT* system was used successfully to demonstrate that *E74* and *E75* are required for follicle development (Buszczak *et al.* 1999), and that *usp* and *E74* are required for GSC maintenance and proliferation (Ables and Drummond-Barbosa 2010). In brief, an *FRT* site lies proximal to a mutation in a gene of interest *in trans* to another *FRT* chromosome arm carrying the corresponding wildtype allele linked to a GFP marker. *FLP* catalyzes mitotic recombination between the *FRT* sites in dividing cells, leading to the formation of clones of homozygous mutant cells in the context of wildtype tissue (Figure 2A). We took advantage of a heat-shock-promoter-driven *FLP* transgene that expresses the FLP recombinase in response to high temperature treatment, and thus mediates recombination in a time-controlled manner. This allowed us to examine the effects of putative ecdysone targets specifically in adult ovarian cells, circumventing the developmental lethality typically associated with ecdysone-responsive genes, and providing information about ovarian cell type- and stage-specific requirements. It should be noted that, although ecdysone is required for events occurring during later stages of oogenesis, such as vitellogenesis and border cell migration (Figure 1C), our screen design precluded identification of genes involved in these processes.

To develop an initial list of putative ecdysone-responsive genes to be screened for roles in oogenesis, we cross-referenced 3505 genes previously identified as candidate ecdysone targets by two genome-wide datasets (Beckstead *et al.* 2005; Gauhar *et al.* 2009) with the BruinFly collection (Chen *et al.* 2005; Call *et al.* 2007). Ecdysone targets were identified by Beckstead and colleagues by microarray comparison of cultured organ explants from wildtype and *EcR* knockdown larvae (Beckstead *et al.* 2005), and by Gauhar and colleagues by DNA Adenine Methyltransferase Identification (DAM-ID) in the ecdysone-responsive embryonic Kc cell line (Gauhar *et al.* 2009). The BruinFly collection comprises over 1000 lethal transposon insertion lines that were each individually recombined with an appropriate *FRT* site for the purpose of analyzing gene function in a cell context-dependent manner (Chen *et al.* 2005; Call *et al.* 2007). We identified 417 BruinFly lines harboring transposon insertions in putative ecdysone response genes. Due to the high efficiency of *FLP*/*FRT*-mediated mitotic recombination that we typically observe in experiments using the *FRT40A* and *FRT82B* chromosome arms, we chose 56 BruinFly lines with mutations in genes located on the left arm of chromosome II or the right arm of chromosome III for screening for potential ovarian phenotypes.

### Genetic mosaic screening reveals a wide variety of candidate ecdysone-responsive genes that function in the ovary

While the initial focus of our screen was to find mutants with defects in GSC maintenance, we observed that many of the lines we tested had defects in other early oogenesis processes. Indeed, surprisingly, more than 65% of the BruinFly lines examined displayed ovarian phenotypes when homozygous clones were present in the germline, the soma, or both (File S1). Ovarian phenotypes, such as defects in germline growth or survival, were often, but not always, associated with stem cell loss. Many of the putative ovarian ecdysone-responsive genes identified in our screen have also been independently identified as regulators of stem cells (43% of genes) or the germline (35% of genes) in recent large scale short hairpin interfering RNA (RNAi) screens (File S1) (Neumuller *et al.* 2011; Yan *et al.* 2014; Zeng *et al.* 2015; Sanchez *et al.* 2016). Protein ANalysis THrough Evolutionary Relationships (PANTHER) gene ontology analysis of molecular function demonstrates that many of the genes identified in our screen produce proteins that function in molecular binding (File S1). Indeed, genes classified as having the molecular function of sequence-specific DNA binding are significantly overrepresented in our dataset of ecdysone-induced ovarian targets; additional targets are classified as having translational regulator activity, enzyme regulatory activity, or receptor activity (File S1). Gene ontology analysis of biological processes shows that most of the genes identified in our screen are associated with development; significant numbers of genes are also associated with RNA metabolic processes, regulation of gene expression, and neurogenesis (File S1). Given the variety of mutant phenotypes uncovered in our screen, we categorized the ecdysone-responsive genes into five classes based on cell lineage requirement (germline *vs.* soma) and mutant phenotype: GSC loss; defects in germline proliferation, growth, or survival; FSC loss; defects in somatic proliferation, growth, or survival; or encapsulation defects (Table 1).

**Table 1:** Summary of mutant ovarian phenotypes revealed by screen

Gene Symbol	Gene Name	Cytology	BruinFly Allele
**Class I: GSC loss**			
* Trn-SR*	*Transportin-Serine/Arginine rich*	23A3	*Trn-SR^KG04870^*
* vkg*	*viking*	25C1	*vkg^k00236^*
* Kr-h1*	*Kruppel homolog 1*	26B5	*Kr-h1^KG00354^*
* Hrb27C*	*Heterogeneous nuclear ribonucleoprotein at 27C*	27C4	*Hrb27C^k02814^*
* Acer*	*Angiotensin-converting enzyme-related*	29D4	*Acer^k07704^*
* CG9305*, *CG6565*	*CG9305*, *CG6565*	34B8	*CG9305^EY01878^*
* CycE*	*Cyclin E*	35D4	*CycE^KG00239^*
* crp*	*cropped*	35F1	*crp^KG08234^*
* Tpr2*	*Tetratricopeptide repeat protein 2*	36A2	*Tpr2^KG08262^*
* VhaSFD*	*Vacuolar H^+^-ATPase SFD subunit*	36A12	*VhaSFD^EY04644^*
* MESR3*	*Misexpression suppressor of ras 3*	36F7	*MESR3^EP2221^*
* Hr39*	*Hormone receptor-like in 39*	39C	*Hr39^Scim^*
* Df31*	*Decondensation factor 31*	39E3	*Df31^k05815^*
* CG12050*	*CG12050*	62B4	*CG12050^KG03759^*
* Droj2*	*DnaJ-like-2*	87E8	*Droj2 ^l(3)87Eg-s2149^*
* trx*	*trithorax*	88B1	*trx^j14A6^*
* Dph5*	*Diphthamide methyltransferase*	94B5	*Dph5^L4910^*
			
**Class II: defects in germline proliferation, growth, or survival**			
* dbe*	*dribble*	21E2	*dbe^k05428^*
* Trn-SR*	*Transportin-Serine/Arginine rich*	23A3	*Trn-SR^KG04870^*
* CG17259*	*CG17259*	23C5	*CG17259^KG03126^*
* FASN1*	*Fatty acid synthase 1*	23C5	*FASN1^EY05632^*
* vkg*	*viking*	25C1	*vkg^k00236^*
* hoip*	*hoi-polloi*	30C5	*hoip^k07104^*
* CycE*	*Cyclin E*	35D4	*CycE^KG00239^*
* Tpr2*	*Tetratricopeptide repeat protein 2*	36A2	*Tpr2^KG08262^*
* VhaSFD*	*Vacuolar H^+^-ATPase SFD subunit*	36A12	*VhaSFD^EY04644^*
* MESR3*	*Misexpression suppressor of ras 3*	36F7	*MESR3^EP2221^*
* CG10341*	*CG10341*	37A1	*CG10341^f07749^*
* CG12050*	*CG12050*	39A1	*CG12050^KG03759^*
* Hr39*	*Hormone receptor-like in 39*	39C	*Hr39^Scim^*
* trx*	*trithorax*	88B1	*trx ^j14A6^*
* CtBP*	*C-terminal Binding Protein*	87D8	*CtBP^KG07519^*
* 14-3-3ε*	*14-3-3ε*	90F10	*14-3-3ε ^j2B10^*
			
**Class III: FSC loss**			
* dbe*	*dribble*	21E2	*dbe^k05428^*
* Trn-SR*	*Transportin-Serine/Arginine rich*	23A3	*Trn-SR^KG04870^*
* vri*	*vrille*	25D4	*vri^k05901^*
* dsf*	*dissatisfaction*	26A1	*dsf^f00109^*
* Hrb27C*	*Heterogeneous nuclear ribonucleoprotein at 27C*	27C4	*Hrb27C^k02814^*
* Acer*	*Angiotensin-converting enzyme-related*	29D4	Acer*^k07704^*
* crol*	*crooked legs*	33A1	*crol^k05205^*
* CG9305*, *CG6565*	*CG9305*, *CG6565*	34B8	*CG9305^EY01878^*
* CycE*	*Cyclin E*	35D4	*CycE^KG00239^*
* crp*	*cropped*	35F1	*crp^KG08234^*
* MESR3*	*Misexpression suppressor of ras 3*	36F7	*MESR3^EP2221^*
* Df31*	*Decondensation factor 31*	39E3	*Df31^k05815^*
* CG12050*	*CG12050*	62B4	*CG12050^KG03759^*
* kra*	*krasavietz*	83B4	*kra^j9B6^*
* CtBP*	*C-terminal Binding Protein*	87D8	*CtBP^KG07519^*
* Droj2*	*DnaJ-like-2*	87E8	*Droj2^l(3)87Eg-s2149^*
* trx*	*trithorax*	88B1	*trx^j14A6^*
* 14-3-3ε*	*14-3-3ε*	90F10	*14-3-3ε ^j2B10^*
* mod(mdg4)*	*modifier of mdg4*	93D7	*mod(mdg4)^L3101^*
* Dph5*	*Diphthamide methyltransferase*	94B5	*Dph5^L4910^*
* pnt*	*pointed*	94E9	*pnt^KG04968^*
* *γ*COP*	*Coat Protein (coatomer)* γ	100C6	γ*COP^KG06383^*
**Class IV: defects in somatic proliferation, growth, or survival**			
* vri*	*vrille*	25D4	*vri^k05901^*
* Kr-h1*	*Kruppel homolog 1*	26B5	*Kr-h1^KG00354^*
* hoip*	*hoi-polloi*	30C5	*hoip^k07104^*
* Dref*	*DNA replication-related element factor*	30F2	*Dref^KG09294^*
* CG9302*, β*COP*	*CG9302*, *Coat Protein (coatomer)* β	34B8	*l(2)k00302*
			*P[EPgy2]EY05965*
* CycE*	*Cyclin E*	35D4	*CycE^KG00239^*
* crp*	*cropped*	35F1	*crp^KG08234^*
* Tpr2*	*Tetratricopeptide repeat protein 2*	36A2	*Tpr2^KG08262^*
* MESR3*	*Misexpression suppressor of ras 3*	36F7	*MESR3^EP2221^*
* CG10341*	*CG10341*	37A1	*CG10341^f07749^*
* Hr39*	*Hormone receptor-like in 39*	39C	*Hr39^Scim^*
* mod(mdg4)*	*modifier of mdg4*	93D7	*mod(mdg4)^L3101^*
* OstStt3*	*Oligosaccharyl transferase 3*	96B19	*OstStt3^j2D9^*
* *γ*COP*	*Coat Protein (coatomer)* γ	100C6	γ*COP^KG06383^*
			
**Class V: encapsulation defects**			
* vri*	*vrille*	25D4	*vri^k05901^*
* kuz*	*kuzbanian*	34C4	*kuz^EY03488^*
* lace*	*lace*	35D2	*lace^k05305^*
* crp*	*cropped*	35F1	*crp^KG08234^*
* MESR3*	*Misexpression suppressor of ras 3*	36F7	*MESR3^EP2221^*
* CG12050*	*CG12050*	39A1	*CG12050^KG03759^*
* CG7800*	*CG7800*	84F4	*CG7800^KG08575^*
* Atp*α	*Na pump α subunit*	93A4	*Atp*α *^j5C7^*

### Class I: mutants affecting GSC maintenance

We have previously demonstrated that ecdysone signaling, at least in part via the transcription factor *E74*, is critical for maintenance of GSC fate (Ables and Drummond-Barbosa 2010); however, it remained unclear what additional ecdysone-responsive genes contribute to GSC maintenance. We therefore focused our screen on identifying mutant alleles with defects in GSC maintenance (Figure 2, B and C). To this end, we collected samples 12 d after clone induction, to allow sufficient time for homozygous mutant GSCs to divide several times, and for non-GSC-derived clones to be cleared from the germarium (Margolis and Spradling 1995). GSCs and their progeny can be easily identified by coimmunofluorescent detection of Hts, a component of the fusome (a germline-specific organelle with a distinctive morphology), and Lamin C, a nuclear envelope protein that is highly expressed in somatic cap cells (the major cellular component of the GSC niche). While both GSCs and their daughters can be recognized by the presence of the fusome, its morphology varies through early germ cell development, and only GSCs possess an anteriorly localized fusome juxtaposed to the interface with cap cells (Lin *et al.* 1994; de Cuevas and Spradling 1998).

In germline clones that arise following *FLP/FRT*-mediated recombination, the homozygous mutant (or wildtype, in case of controls) GSC and all of its progeny are recognizable by the absence of the GFP marker (Figure 2A). In control mosaic germaria, where all cells are genetically wild type, GFP-negative GSCs and their progeny typically coexist (Figure 2B). In contrast, frequent instances of GFP-negative cystoblasts/cysts present in the absence of a GFP-negative GSC were observed in many mutant mosaic germaria (Figure 2C, for example). In these germaria, a GFP-negative GSC was generated (as evidenced by their progeny), but was presumably lost due to cell death or premature differentiation over the course of the experiment (*i.e.*, a “GSC loss” phenotype). We therefore scored GSC loss in two ways: in Method I, we quantified the percentage of germline mosaic germaria with a GSC loss phenotype; in Method II, we quantified the percentage of all germaria carrying a GFP-negative GSC (Table S2).

We observed statistically significant GSC loss in a total of 17 transposable element insertion lines, including six impacting genes previously identified as regulators of stem cell fate or function in large-scale RNAi screens (Neumuller *et al.* 2011; Yan *et al.* 2014; Zeng *et al.* 2015; Sanchez *et al.* 2016): *Cyclin E* [*CycE*, described previously in detail (Ables and Drummond-Barbosa 2013)]; *Heterogeneous nuclear ribonucleoprotein at 27C* (*Hrb27C*); *Transportin-Serine/Arginine rich* (*Trn-SR*); *CG12050*; *DnaJ-like-2* (*Droj2*); and *CG9305* (File S1 and Table S2). In particular, we observed a very high rate of GSC loss (63.2%; Table S2) in mosaic germaria harboring a strong loss-of-function allele of *Hrb27C* (Figure 2C). Hrb27C (also known as Hrp48) is an abundant protein required in the developing oocyte for the proper localization of critical polarity-determining transcripts (Matunis *et al.* 1992a, 1992b; Goodrich *et al.* 2004; Huynh *et al.* 2004; Yano *et al.* 2004). Although previous studies showed that *Hrb27C*-deficient germline cysts fail to develop past early stages in oogenesis (Yano *et al.* 2004), little is known about the role of *Hrb27C* as a potential regulator of GSC maintenance. Our results are consistent, however, with results from a recent large-scale RNAi screen that identified other heterogeneous ribonucleoproteins as critical determinants of stem cell self-renewal (Yan *et al.* 2014). To confirm that *Hrb27C* is ecdysone-responsive, we used quantitative reverse-transcriptase PCR to measure *Hrb27C* mRNA levels in whole ovaries from *EcR^ts^* mutant females, which display decreased ecdysone signaling (Carney and Bender 2000; Ables and Drummond-Barbosa 2010). Indeed, we observed a statistically significant reduction in *Hrb27C* levels in *EcR^ts^* mutant ovaries (Figure S1), suggesting that ecdysone signaling is required for proper *Hrb27C* expression. Additional characterization of the role of *Hrb27C* in GSCs will be detailed in a future manuscript (D.S.F. and E.T.A., in preparation).

**Figure 3:**
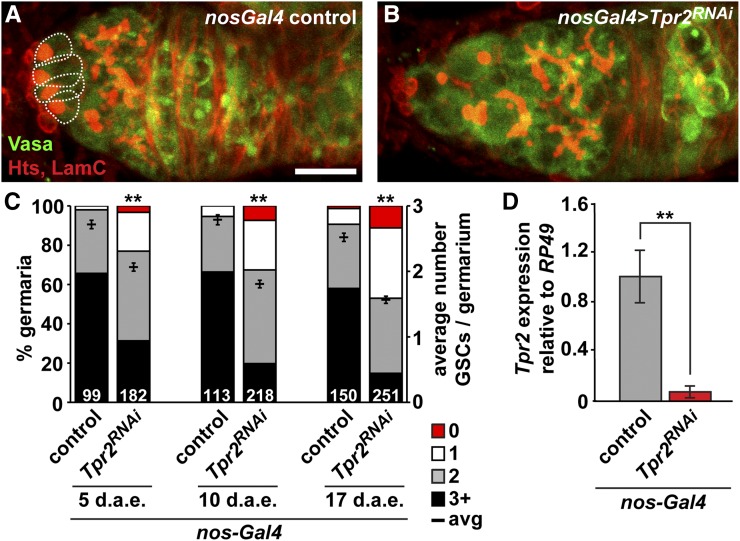
*Tpr2* is required for GSC maintenance. (A–B) Maximum intensity projections of *nos-Gal4* control (A) or *nos-Gal4 > UAS-Tpr2^RNAi^* knockdown (B) germaria labeled with anti-Vasa (green; germ cells), anti-Hts (red; fusomes and follicle cell membranes), and anti-LamC (red; nuclear envelope of cap cells). Dotted lines demarcate GSCs. Scale bar, 10 µm. (C) Frequencies of germaria containing 0 (red), 1 (white), 2 (gray), or 3 or more (black) GSCs per germarium (left *y*-axis), and average number of GSCs per germarium (right *y*-axis) in *nos-Gal4* control or *nos-Gal4 > UAS-Tpr2^RNAi^* knockdown females at 5, 10, and 17 d after eclosion (d.a.e.). The number of germaria analyzed is shown inside bars. (D) Relative expression of *Tpr2* transcripts in *nos-Gal4* control and *nos-Gal4 > UAS-Tpr2^RNAi^* ovaries. Bars indicate average relative quantitative reverse-transcriptase PCR ΔΔCq ratios from three biological replicates, normalized to reference gene *rp49* expression and to *nos-Gal4* biological controls. Error bars, mean ± SEM. ** *P* < 0.0001; Student’s two-tailed *t*-test.

We also identified a strong GSC loss phenotype in a loss-of-function allele corresponding to *Tetratricopeptide repeat protein 2* (*Tpr2*), a previously undescribed ecdysone-responsive gene (Table S2). The biological functions of *Tpr2* are virtually unknown, making it an intriguing candidate for further study. Tpr2 is predicted to contain a tetratricopeptide repeat region, which mediates protein–protein interactions, and a DNAJ domain, characteristic of molecular chaperones, suggesting roles in cell cycle regulation, transcriptional control, and/or protein folding. Interestingly, *Tpr2* has been identified in several genome-wide studies, as both a regulator of fecundity, and a target of ecdysone signaling (Beckstead *et al.* 2005; Gan *et al.* 2010; Durham *et al.* 2014). *Tpr2* transcripts are highly enriched in the adult ovary (Graveley *et al.* 2011), and *Tpr2* mRNA levels are significantly decreased concomitantly with decreased ecdysone signaling (Figure S1). As very few transposable element insertion alleles are available for *Tpr2*, we sought to verify a role for this ecdysone target gene in GSCs by using RNAi to specifically reduce *Tpr2* function in developing germ cells via the *UAS/Gal4* system (Ni *et al.* 2011). We crossed an available germline-compatible *Tpr2* RNAi line (Ni *et al.* 2011) to the germline-specific *nos-Gal4* driver (Rørth 1998; Van Doren *et al.* 1998), and counted the number of GSCs per germarium in adult females at 5, 10, and 17 d after eclosion (Figure 3). In contrast to controls, we detected a significant decrease in GSC number in germline-specific *Tpr2* knockdown germaria at all timepoints examined (Figure 3C). By 17 d after eclosion, germaria were dramatically reduced in size (Figure 3B), and many were devoid of GSCs (Figure 3C). Consistent with our mosaic analysis, these results support the hypothesis that *Tpr2* is directly required in GSCs for proper maintenance. Interestingly, we also noted that germline-specific reduction of *Tpr2* via RNAi resulted in dramatically decreased levels of *Tpr2* transcripts in whole ovaries (Figure 3D), suggesting that *Tpr2* expression may be restricted to the germline.

We also identified a mutant allele of the nuclear hormone receptor, *Hormone receptor-like in 39* (*Hr39*), as having statistically significant GSC loss (Table S2). A variety of nuclear hormone receptors have been genetically linked to the ecdysone signaling pathway, many as targets that are upregulated in response to ecdysone (King-Jones and Thummel 2005; Yamanaka *et al.* 2013); however, only a few have thus far been demonstrated to function in oogenesis (Buszczak *et al.* 1999; Carney and Bender 2000; Ables and Drummond-Barbosa 2010; Sun and Spradling 2012; Ables *et al.* 2015). Hr39 has previously been implicated in reproductive tract development; specifically, it is required for the formation of spermathecae and the three-cell secretory units (Allen and Spradling 2008; Sun and Spradling 2012). Hr39 is also thought to regulate axon pruning in larval mushroom bodies via an interaction with EcR and Ftz-f1, a related nuclear hormone receptor (Boulanger *et al.* 2011). *Hr39* was therefore an exciting candidate regulator of the ecdysone signaling pathway in GSCs. Upon further testing, however, we were unable to verify a role for *Hr39* in the control of GSC maintenance (Figure S2). We examined ovary morphology, and quantified GSC number in four previously characterized *Hr39* loss-of-function alleles in *trans* to a deficiency that uncovers all of the *Hr39* locus (Allen and Spradling 2008; Boulanger *et al.* 2011; Sun and Spradling 2012). While we observed the previously described phenotypes (*i.e.*, decreased egg laying, egg retention, and decreased number of spermathecae), we found no changes in either germarium morphology (Figure S2, A–F) or GSC number (Figure S2G) in the absence of *Hr39*. We verified that the *P* element insertion in the BruinFly allele used in our mosaic analysis indeed mapped to the 5′ untranslated region of the *Hr39* locus (G.H.H., E.T.A., and D.D.-B., unpublished data), but concluded that the strong GSC loss phenotype observed in our screen must be due to the presence of a second site mutation in the BruinFly line, which is also likely responsible for its lethality. Our results therefore suggest that *Hr39* is not required for GSC maintenance or function.

### Class II: mutants affecting early germline proliferation, growth, or survival

**Figure 4:**
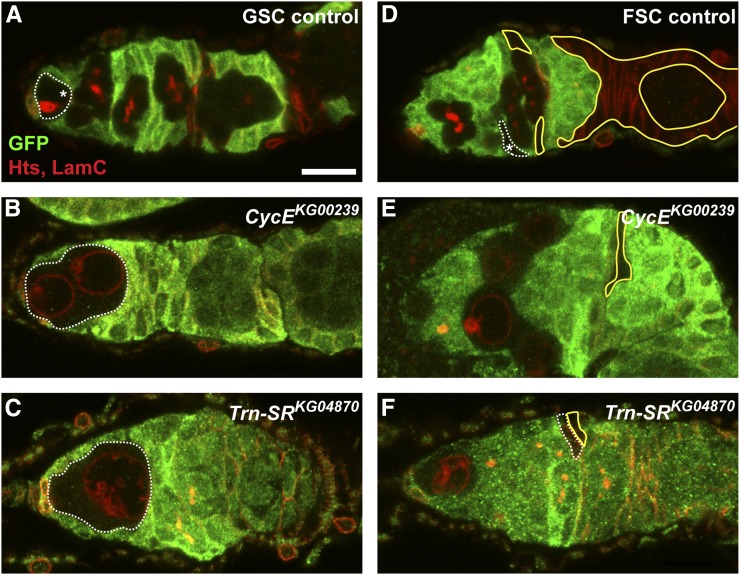
Putative ecdysone-responsive genes are required for proper progression through the cell cycle in GSCs and FSCs. (A–F) Representative mock mosaic control (A, D) or ecdysone target gene mutant (B, C, E, F) germaria labeled with anti-GFP (green), anti-Hts (red; fusomes and follicle cell membranes), and anti-LamC (red; nuclear envelope of cap cells). Dotted lines demarcate GFP-negative GSCs or FSCs; asterisks denote wild-type stem cells; solid yellow lines demarcate GFP-negative daughter follicle cells. Scale bar, 10 µm.

**Figure 5:**
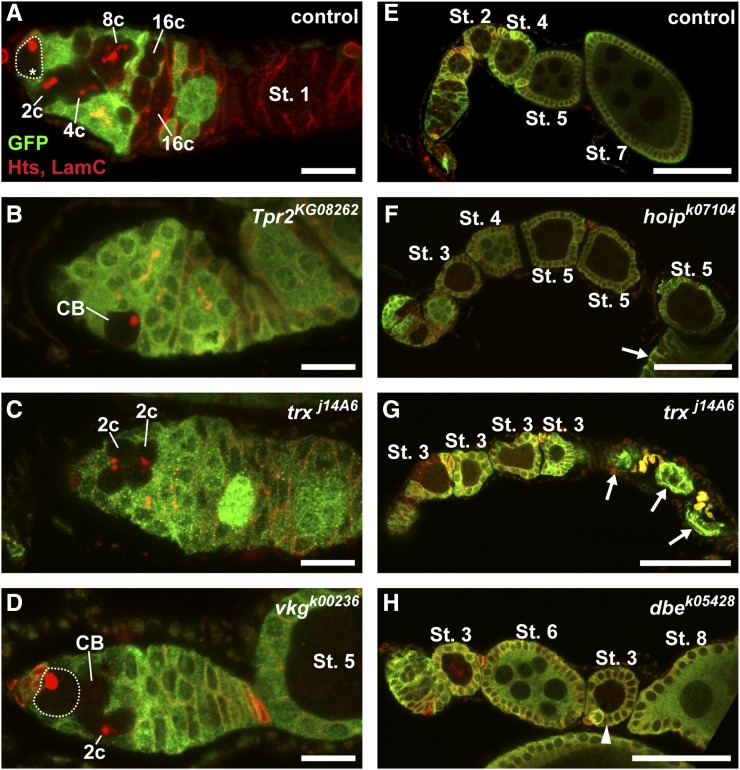
Putative ecdysone-responsive genes are required in the germline for proper cyst growth and survival. (A–H) Representative mock mosaic control (A, E) or ecdysone target gene mutant (B–D, F–H) germline-mosaic germaria (A–D) or germline-mosaic ovarioles (E–H) labeled with anti-GFP (green), anti-Hts (red; fusomes and follicle cell membranes), and anti-LamC (red; nuclear envelope of cap cells). Dotted lines demarcate GFP-negative GSCs; asterisk denotes wildtype GSC; arrows indicate dying follicles; arrowhead indicates small follicle with a mutant cyst in between two larger wildtype follicles. Oogenesis stages determined (except in the case of dying follicles) by follicle size and nurse cell nuclear morphology, as described (Spradling 1993). Scale bar, 10 µm (A–D) or 50 µm (E–H).

In addition to GSC loss, our screen revealed new candidate ecdysone-responsive genes required for the proliferation, growth, or survival of the early germline (Figure 4 and Figure 5). Most prominent were a variety of mutants with clear cell cycle defects in GSCs and their immediate daughters (Figure 4). For example, loss of the well-known cell cycle regulator *CycE* in GSCs and cystoblasts causes a block in cell cycle progression, such that cells are arrested in G1, fail to divide, and grow to unusually large sizes (Figure 4B) (Ables and Drummond-Barbosa 2013). Similar phenotypes were observed in mosaic germaria harboring a transposon insertion in the *Trn-SR* locus (Figure 4C). In *Trn-SR* mutant germaria, large single GFP-negative cells were frequently detected in the niche; these cells lacked an anteriorly localized fusome, and expressed high levels of the nuclear membrane protein Lamin C. Further, nuclei in *Trn-SR* mutant cells appeared to have many folds or deformations of the nuclear membrane. Quantitative mRNA analysis confirmed that *Trn-SR* expression is dependent on proper ecdysone signaling (Figure S1). While *Drosophila* Trn-SR has not been well characterized, it shares considerable sequence similarity with mammalian Importin β proteins—a conserved family of transport proteins that mediate the intranuclear and nuclear-cytoplasmic shuttling of RNA-binding proteins (Allemand *et al.* 2002). Mammalian Importin β proteins have recently been associated with the regulation of mitosis (Forbes *et al.* 2015), and our data suggest that this function may be conserved in *Drosophila* GSCs and their progeny. Intriguingly, *Trn-SR* was also identified as a regulator of neuroblast self-renewal (Neumuller *et al.* 2011), suggesting a conserved role in stem cell function.

We also observed defects in early germ cells or in developing 16-cell cysts consistent with roles for some candidate ecdysone-responsive genes in cell cycle progression, cell growth, or cell survival. During normal oogenesis, with each round of GSC division, one daughter cell (the cystoblast) is produced that, in turn, divides four times with incomplete cytokinesis, forming 2-, 4-, 8-, and 16-cell cysts (Figure 1B). In control mock mosaics, GFP-negative germ cells in the germarium can be observed at all of these stages of development (Figure 5A). In contrast, we found that *Tpr2* mutant mosaic germaria rarely contained GFP-negative multicellular germline cysts, indicative of defects in the differentiation, proliferation, growth, and/or survival of early germ cells in relation to surrounding wild type cells (Figure 5B). Germline-specific RNAi-mediated knockdown of *Tpr2*, however, demonstrated that multicellular cysts can form when *Tpr2* levels are more mildly decreased (Figure 3B). Similar phenotypes were observed in mosaic germaria carrying mutations in the histone methyltransferase *trithorax* (*trx*; Figure 5C) and *viking* (*vkg*), encoding a *Drosophila* Collagen IV subunit (Figure 5D). *trx* and *vkg* mutant mosaic germaria displayed GFP-negative cystoblasts and 2-cell cysts, but lacked more differentiated 4-, 8- or 16-cell cysts, suggesting a block in differentiation, and/or increased cell death following the second mitotic division.

Our results indicating cell autonomous GSC loss (Table S2) and defective germline proliferation and/or differentiation (Figure 5D) phenotypes in a known mutant of *vkg* are somewhat surprising. Collagen IV and a variety of integrin subunits are detected in the basement membrane adjacent to the adult GSC niche (particularly around cap cells), and in the posterior half of the germarium adjacent to the FSCs and prefollicle cells (O’Reilly *et al.* 2008; Wang *et al.* 2008; Van De Bor *et al.* 2015). Collagen IV is thought to be deposited in the GSC niche by hemocytes during larval development (Van De Bor *et al.* 2015); indeed, germ cells do not produce detectable levels of *vkg* mRNA in adults (Van De Bor *et al.* 2015). Thus, while previous studies do not rule out a direct role for *vkg* in GSC maintenance or germ cell proliferation, these data may suggest the presence of a second site mutation in the *vkg* BruinFly allele, similar to our findings for the *Hr39* BruinFly allele. Additional studies will be necessary to test for a cell-autonomous role for *vkg* in the germline, and to reveal an unknown, yet potentially important, regulator of early germline development disrupted by the second site mutation.

We also observed a variety of mutants with defects in cyst growth or survival outside of the germarium. In control germline-mosaic ovarioles (Figure 5E) where all germ cells are GFP-negative (yet the surrounding follicle cells are largely GFP-positive), a normal progression of increasingly larger, more developed, follicles can be observed, as expected (Figure 1A). Germline-mosaics harboring a transposable element in the *hoi-polloi* (*hoip*) locus progressed normally through the first five follicular stages (Figure 5F), but appeared to arrest and degenerate at the stage 5/6 transition (arrow in Figure 5F). Similarly, *trx* mutant germline mosaics exhibited an accumulation of stage 3 follicles, followed by degenerating follicles (Figure 5G). Furthermore, we observed *dribble* (*dbe*) mutant cysts in follicles with characteristic stage 3 size and morphology, but located posteriorly to much more developed wild type follicles (Figure 5H). *hoip* and *dbe* encode RNA-binding proteins, and are essential for ribosome biogenesis via roles in rRNA processing (Chan *et al.* 2001; Murata *et al.* 2008). Ecdysone signaling is known to regulate cyst growth and survival (Buszczak *et al.* 1999; Carney and Bender 2000; Ables *et al.* 2015); these results suggest that *hoip*, *trx*, and *dbe* may mediate this response.

### Class III: mutants affecting FSC maintenance

Because the *FLP*/*FRT* mosaic recombination system functions in any mitotically active cell, we were able to test whether putative ecdysone-responsive genes function in FSCs and their daughter cells in parallel to our germline analysis. As for the germline lineage, germaria containing GFP-negative FSCs along with their descendants are readily observed in control mosaics (Figure 2D). We therefore scored FSC loss using the same methodology as applied to the germline lineage (Laws and Drummond-Barbosa 2015). Although ecdysone signaling has not yet been directly demonstrated to have a cell-autonomous role in maintenance of FSCs, we found a large number of putative ecdysone-responsive gene mutants with a FSC loss phenotype (Table S3). For example, *pointed* (*pnt*) mutant germaria were frequently observed to have small GFP-negative prefollicle or early follicle cell clones in the absence of a GFP-negative FSC (Figure 2E). In total, we observed statistically significant FSC loss in 22 mutant alleles (Table S3), including several that also displayed significant GSC loss (Table S2). Among these, *CycE* and *krasavietz* (*kra*) have been previously demonstrated to regulate FSCs and/or their daughters (Wang and Kalderon 2009; Jia *et al.* 2015).

We observed a variety of ovarian defects in *trx* mutant mosaic ovarioles, including FSC loss (Table S3). *trx* is the founding member of the Trithorax group (TrxG) of chromatin regulators, a diverse group of proteins that promote heritable states of gene expression by regulating a variety of developmental master regulatory genes (Kingston and Tamkun 2014). TrxG proteins play important roles in the maintenance of cell fate; thus, it is noteworthy that we observe ovarian stem cell loss in *trx* mutant mosaic ovarioles. While no direct link has previously been established between *trx* and *EcR*, chromatin modifications frequently accompany ecdysone-responsive gene expression (Yamanaka *et al.* 2013). Two related TrxG methyltransferases, encoded by *absent*, *small*, or *homeotic discs 2* (*ash2*), and *trithorax-related* (*trr*), function as EcR coactivators, modulating chromatin structure at ecdysone-responsive enhancers (Sedkov *et al.* 2003; Carbonell *et al.* 2013). Further, we and others have shown that ecdysone signaling functionally interacts with the Nucleosome Remodeling Factor (NURF) complex to promote target gene expression (Badenhorst *et al.* 2005), and maintenance of the GSC fate (Ables and Drummond-Barbosa 2010). Thus, it appears likely that ecdysone signaling broadly interacts with the cellular chromatin modifying machinery to regulate gene expression and maintenance of cell fate. While future experiments will be necessary to test conclusively the role of *trx* and other TrxG proteins downstream of ecdysone in the ovary, our data suggest that *trx* could be an important mediator of ecdysone signaling in ovarian stem cells.

### Class IV: mutants affecting early somatic proliferation, growth, or survival

Our screen also provided us with the opportunity to test the function of putative ecdysone-responsive genes in the growth and proliferation of early follicle cells. We quickly noted, however, that it was difficult to determine if *FRT40A*-containing mutants affected later follicle cell development, as many had phenotypic defects reminiscent of those resulting from mutations in *lethal (2) giant larvae* [*l(2)gl*]. Previous studies have noted a high frequency of *l(2)gl* alleles in the Bruinfly *FRT40A* collection, likely because many of these stocks harbor terminal deletions of the left arm of the second chromosome, including the *l(2)gl* locus (Roegiers *et al.* 2009). Despite the confounding results due to the presence of *l(2)gl* mutations, we observed a small number of ecdysone-responsive gene mutants with defects in early somatic proliferation, growth, or survival (Figure 4). For example, prefollicle cells carrying mutations in *CycE* (Figure 4E) and *Trn-SR* (Figure 4F), described above for their germline phenotypes, failed to produce daughter cells as frequently as mock mosaic controls (Figure 4D). Intriguingly, in contrast to mutant germ cells, neither *CycE* nor *Trn-SR* mutant follicle cells grew excessively. This may suggest that the primary role for *CycE* and *Trn-SR* is in the regulation of cell cycle progression, and that the regulation of cell size is fundamentally different between germ cells and somatic prefollicle cells.

### Class V: mutants affecting cyst encapsulation

In the posterior of the germarium, follicle cells encapsulate 16-cell germline cysts and subsequently pinch them away from the germarium, forming individual follicles (Figure 1). Each follicle is composed of a monolayer of follicle cells surrounding a germline cyst, and follicles are joined together by stalk cells (a specialized subpopulation of follicle cells). While a few studies have identified mutants with defects in encapsulation, the cell biology of encapsulation is not well understood. In the prevailing model, escort cells in the germarium extend long cellular processes that wrap individual germline cysts as they divide, and guide cysts to new follicle cells [produced by FSCs; (Kirilly *et al.* 2011; Morris and Spradling 2011)]. Follicle cells then migrate centripetally between 16-cell cysts as the cysts are pushed posteriorly, forming a follicular cuboidal epithelium around each cyst (King 1970; Horne-Badovinac and Bilder 2005). Lastly, follicle cells anterior to the budding follicle interleaf to form a stalk, separating the new follicle from the germarium. Follicle cells differentiate into polar, stalk, and main-body follicle cell subtypes concomitant with their migration (Horne-Badovinac and Bilder 2005; Assa-Kunik *et al.* 2007).

Several signaling pathways control follicle cell differentiation and cyst encapsulation. Initial specification of follicle cells is controlled by the antagonistic actions of Hedgehog signaling and Eyes Absent, which represses Castor to divide main-body follicle cell precursors from polar/stalk cell precursors (Chang *et al.* 2013). Notch/Delta (initiated by Delta ligands produced by the germline) and JAK/STAT signaling then work in combination to specify polar and stalk cell fates (Horne-Badovinac and Bilder 2005; Assa-Kunik *et al.* 2007). The precise mechanisms by which follicle cells recognize and envelope an individual cyst are largely unknown, but are clearly tied to follicle cell differentiation: for example, mutations in both Notch/Delta and JAK/STAT signaling result in encapsulation defects (Lopez-Schier and St Johnston 2001; Baksa *et al.* 2002; McGregor *et al.* 2002).

**Figure 6:**
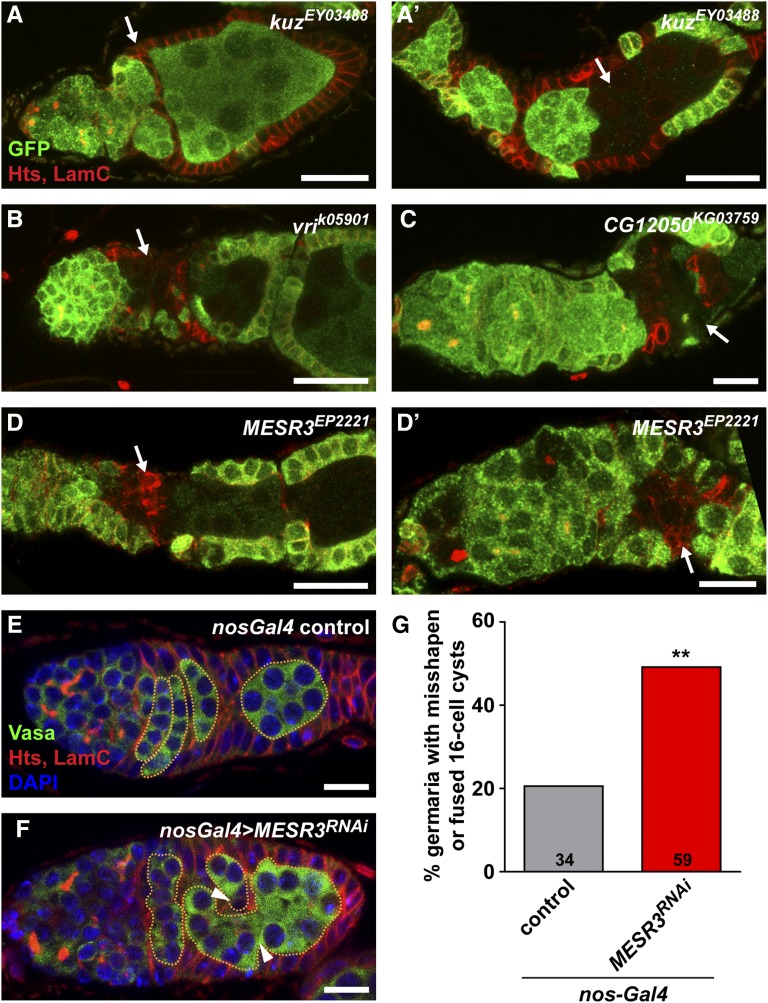
Putative ecdysone-responsive genes are required in the germline and the soma for the proper encapsulation of germline cysts by follicle cells. (A–D’) Representative ecdysone-responsive gene mutant ovarioles labeled with anti-GFP (green), anti-Hts (red; fusomes and follicle cell membranes), and anti-LamC (red; nuclear envelope of cap cells). The anterior end of each ovariole, including the germarium and the first one to two follicles, is shown. Arrows indicate follicular encapsulation defects. (E–F) *nos-Gal4* control (E) or *nos-Gal4 > UAS-MESR3^RNAi^* (F) germaria labeled with anti-Vasa (green; germ cells), anti-Hts (red; fusomes and follicle cell membranes), anti-LamC (red; nuclear envelope of cap cells), and DAPI (blue; nuclei). Dotted lines demarcate 16-cell germline cysts in the posterior of the germarium. Arrowheads indicate overlapping cysts, giving the appearance of a cyst fusion. Scale bars, 10 µm (C, D′, E–F) or 20 µm (A–B, D). (G) Percentage of *nos-Gal4* control or *nos-Gal4 > UAS-MESR3^RNAi^* knockdown germaria with misshapen or “fused” cysts in the posterior (Region 3) of the germarium. The numbers of germaria analyzed are shown inside bars. ** *P* < 0.01; Chi-square test.

Loss-of-function mutants of several ecdysone signaling-related genes, including *E74*, *E75*, *EcR*, and *ecd* display defects in follicle encapsulation (Buszczak *et al.* 1999; Carney and Bender 2000; Morris and Spradling 2012; E.T.A. and D.D.-B., unpublished data). Defects generally include gaps in the follicular epithelium, supernumerary germ cells per follicle, and/or cell death. We observed a variety of putative ecdysone-responsive gene mutant mosaic ovarioles with similar phenotypes (Figure 6). For example, *kuzbanian* (*kuz*) mosaic ovarioles with somatic clones (Figure 6A) failed to separate into individual follicles, and those with germline clones (Figure 6A′) contained multiple germline cysts in a single follicle. Consistent with the known role of *kuz* as a regulator of Notch/Delta signaling, these results phenocopy *Notch* and *Delta* somatic and germline mutant clones, respectively (Pan and Rubin 1997; Qi *et al.* 1999; Lopez-Schier and St Johnston 2001). Notch signaling has been previously demonstrated to transcriptionally activate *br*, an ecdysone-inducible gene (Jia *et al.* 2014); our data suggest that additional functional interactions between the Notch and ecdysone pathways may control follicular encapsulation.

Other mutant mosaics, including *vrille* (*vri*; Figure 6B), *CG12050* (Figure 6C), and *Misexpression suppressor of ras 3* (*MESR3*; Figure 6, D and D′) had a distinct phenotype: follicles frequently failed to bud away from the germarium, or displayed gaps in the follicular epithelium. Abnormal individualization of follicles accompanied mutant cysts surrounded by wildtype follicle cells, but stronger phenotypes were observed in follicles with both GFP-negative follicle cells and cysts. For example, *MESR3* mutant follicle cells adjacent to mutant cysts had irregular cell shapes, and failed to integrate normally into the follicular epithelium (Figure 6, D and D′), but it was unclear whether abnormal encapsulation was the result of primary defects in the germline or the soma. To test whether *MESR3* was required in the germline for proper encapsulation, we analyzed germarium structure in ovarioles harboring germline-specific *MESR3^RNAi^* (Figure 6, E–G). Coimmunofluorescent detection of Vasa, a germ cell-specific protein, and Hts, expressed in both germline fusomes and the plasma membrane of all follicle cells, allowed for easy identification of germ cells within the developing follicular epithelium. In the vast majority of *nos-Gal4* controls, lens-shaped 16-cell cysts were arranged perpendicularly to the germarium anterior-posterior axis, separated by follicle cells (Figure 6, E and G). Similar to observations made from transmission electron micrographs (King 1970), we noted that while most of the lens-shaped cysts were clearly arranged in a single-file order (Figure 6E), some germaria were observed with overlapping cysts, giving the appearance of cyst fusions (Figure 6G). In contrast, 49% of *nos-Gal4 > MESR3^RNAi^* germaria (Figure 6G) displayed defects in cyst shape and encapsulation, including increased numbers of “fused” cysts (arrowheads in Figure 6F), misshapen or misoriented cysts, and improper follicle cell centripetal migration. The average overall number of 16-cell cysts in the posterior germarium (Region 3), however, remained unchanged (control = 4.5 cysts, *n* = 34 germaria; *nos-Gal4 > MESR3^RNAi^* = 4.6 cysts, *n* = 59 germaria), suggesting that while wild-type prefollicle cells appear to migrate more slowly to *MESR3* mutant cysts, most cysts are eventually encapsulated properly. While these data suggest a germline-autonomous role for *MESR3* in cyst encapsulation, we cannot conclusively rule out roles for *MESR3* in the soma. Indeed, we failed to detect significant decreases in *MESR3* transcript level in *nos-Gal4 > MESR3^RNAi^* whole ovaries (Figure S3A), suggesting that significant expression is contributed by somatic cells. Likewise, we did not detect statistically significant reductions in GSC number in females with germline-specific knockdown of *MESR3* (Figure S3B), despite evidence of GSC loss in our mosaic analysis (Table 1). As these results may reflect insufficient RNAi knockdown in the germline, further testing will be necessary to confirm whether *MESR3* is required for oogenesis. MESR3 is predicted to contain a pleckstrin homology-like domain, suggesting association with phosphorylated membrane lipids, and has been genetically associated with negative regulation of Ras signal transduction (Huang and Rubin 2000). Further, since expression of *MESR3* in the ovary is dependent on proper ecdysone signaling (Figure S1), additional study on the roles of *MESR3* as an ecdysone response gene in oogenesis is warranted.

### Conclusions and potential for future studies

In this study, we identify 39 putative ecdysone-responsive genes that control various cell biological processes during oogenesis, including stem cell maintenance and follicle growth and survival. Many of the genes we identified have been independently verified in recent large-scale RNAi screens, suggesting that these are *bona fide* regulators of oogenesis. Further, the phenotypes we observe in the putative ecdysone-responsive gene mutants are very reminiscent of those of known ecdysone signaling mutants, supporting the idea that a broad network of ovarian factors is regulated by the actions of ecdysone. Importantly, due to the limitations of the BruinFly mosaic approach, each of the genes identified in our screen will require additional experimental testing to confirm the phenotypes we observed. Not all of the BruinFly lines result in null mutations, and some may have background mutations or phenotypes due to transposable element insertion in between two genes or in undescribed gene regulatory regions. Indeed, we did not observe any defects in early oogenesis in a transposon insertion allele of the *brain tumor* locus (File S1), despite its known role in early germ cell differentiation (Harris *et al.* 2011), likely because the BruinFly allele does not result in substantial *brain tumor* loss-of-function. Many of the BruinFly lines, however, are the only known mutant alleles available for some genes, necessitating the isolation of new genetic mutants. CRISPR/Cas9 systems for the creation of novel, precise genetic mutants will prove invaluable for future studies (Xu *et al.* 2015).

The hierarchical model of ecdysone signaling predicts that a relatively small number of early-response genes are directly activated by the transcriptional activity of the ecdysone receptor complex, and that tissue-specific responses to the hormone are generated largely by the differential activity of the early response genes, rather than the ecdysone receptor complex itself (Ashburner 1974; King-Jones and Thummel 2005; Yamanaka *et al.* 2013). Indeed, the two genome-wide datasets from which we identified putative ecdysone targets likely represent a mixture of genes directly bound by the EcR/Usp heterodimer and those indirectly regulated by ecdysone activation (*i.e.*, direct targets of an ecdysone-inducible transcription factor, such as E74 or E75). To more specifically examine whether the genes revealed in our screen are direct targets of ecdysone signaling, we compared publically available DNaseI Hypersensitivity sequencing (DHS-seq), Self-Transcribing Active Regulatory Region sequencing (STARR-seq), and EcR Chromatin Immunoprecipitation sequencing (ChIP-seq) results (Roy *et al.* 2010; Negre *et al.* 2011; Shlyueva *et al.* 2014b; Slattery *et al.* 2014) at specific candidate gene loci for evidence of EcR-dependent activation or repression (Figure S4 and Figure S5). Each method analyzes different biochemical properties characteristic of actively regulated enhancers; for example, DHS-seq identifies DNaseI-accessible, nucleosome-free regions of DNA (a common property of active enhancers), whereas ChIP-seq identifies regions of DNA directly bound by the transcription factor (Shlyueva *et al.* 2014a). Functional enhancers are most likely to be found where there is agreement between the methods (*i.e.*, a segment of DNA contains a “peak” in each method). We found complementary evidence across multiple platforms that four candidate genes, *crooked legs* (*crol*; Figure S4A), *Hrb27C* (Figure S4C), *vrille* (vir; Figure S5A), and *cropped* (*crp*; Figure S5B) are directly regulated by EcR. For example, we identified constitutively open chromatin regions in the 5′ untranslated region and second intron of the *crol* locus, and constitutive activation of an enhancer in an ovarian somatic cell line (OSC) in the second intron (Figure S4A). This enhancer was also bound by EcR in pupal stages. The presence of multiple EcR binding sites and ecdysone-responsive enhancers within introns of target genes has been recently recognized as a predominant feature of ecdysone-responsive genes (Bernardo *et al.* 2014; Shlyueva *et al.* 2014b). These data support the strong functional interactions between *EcR* and *crol* previously reported (D’Avino and Thummel 1998). While further experiments are necessary, this analysis suggests that several of the genes identified in our study may be direct targets of EcR in ovarian cells.

In contrast, the evidence for direct regulation of *Tpr2* and *MESR3* is less clear (Roy *et al.* 2010; Negre *et al.* 2011; Shlyueva *et al.* 2014b; Slattery *et al.* 2014). The *Tpr2* locus was bound by EcR in pupae, but clear regions of enhancer activity in the presence or absence of ecdysone were absent (Figure S4B). The *MESR3* locus was also bound by EcR in pupae, and discrete ecdysone-sensitive enhancers were evident in the first and third introns; however, the EcR binding sites do not align with the enhancers (Figure S5C). Intriguingly, the ecdysone-responsive enhancers appeared to be repressed in the presence of ecdysone, indicating that *MESR3* may be negatively regulated by EcR. These data suggest that either EcR directly regulates *Tpr2* and *MESR3* specifically in ovarian cells, or that these loci are indirectly regulated by ecdysone signaling, potentially through an ecdysone-inducible transcription factor. Indeed, a recent, comprehensive study of the ecdysone transcriptional response in 41 different cell lines estimated that more than 90% of ecdysone-responsive genes function in only a small subset of distinct cell types (Stoiber *et al.* 2016). It is interesting, therefore, to note how many putative ovarian ecdysone-responsive genes identified in our study (previously identified as ecdysone-responsive in other precursor cells) yielded phenotypes in precursor cells in both the germline and soma: tissues of very different developmental origins, but at roughly similar states of differentiation. We speculate that the epigenetic status of the chromatin in different stages of differentiation may be a common feature dictating a cell’s response to the ecdysone signal.

Our study provides a new framework within which to understand the molecular underpinnings of the ecdysone response in ovarian cells. Future studies aimed at more deeply characterizing the molecular function and relationship to ecdysone signaling of each of the identified targets, particularly in ovarian stem cells, will broaden our understanding of how hormonal signals regulate cell fate and proliferation.

## Supplementary Material

Supplemental Material
