# Supplementary material for: A Genetic Mosaic Screen Reveals Ecdysone-Responsive Genes Regulating Drosophila Oogenesis
Source: G3 (Bethesda). 2016 May 24;6(8):2629–42. doi: 10.1534/g3.116.028951 (PMC4978916; doi:10.1534/g3.116.028951)
Supplement: Supplemental Material [file supp_6_8_2629__index.html]

A Genetic Mosaic Screen Reveals Ecdysone-Responsive Genes Regulating Drosophila Oogenesis — Supplemental Material 

# A Genetic Mosaic Screen Reveals Ecdysone-Responsive Genes Regulating *Drosophila* Oogenesis

## Supplemental Material for Ables, *et al*, 2016

**Files in this Data Supplement:**

- File S1 - This file contains the full legends for all Supplemental Material files. (.pdf, 1,363 KB)
- Table S1 - Complete list of screened ecdysone-responsive candidate genes, including gene identifiers, gene ontology enrichment analyses, and comparisons to other screens. (.xlsx, 38 KB)
- Table S2 - Quantification of GSC loss in screened mutants. (.docx, 20 KB)
- Table S3 - Quantification of FSC loss in screened mutants. (.docx, 20 KB)
- Figure S1 - Ovarian expression of putative ecdysone response genes requires optimal ecdysone signaling. (.tif, 309 KB)
- Figure S2 - *Hr39* is not required for GSC maintenance. (.tif, 6,703 KB)
- Figure S3 - Germline-specific knockdown of *MESR3* does not result in decreased *MESR3* transcript levels in whole ovaries or significant reduction in GSC number. (.tif, 367 KB)
- Figure S4 - Ecdysone-responsive genes with ovarian phenotypes may be direct targets of EcR. (.tif, 511 KB)
- Figure S5 - Ecdysone-responsive genes with ovarian phenotypes may be direct targets of EcR. (.tif, 526 KB)
