# Supplementary material for: A Genetic Mosaic Screen Reveals Ecdysone-Responsive Genes Regulating Drosophila Oogenesis
Source: G3 (Bethesda). 2016 May 24;6(8):2629–42. doi: 10.1534/g3.116.028951 (PMC4978916; doi:10.1534/g3.116.028951)
Supplement: Supplemental Material [file supp_g3.116.028951_FileS1.pdf]

SUPPLEMENTAL INFORMATION

A genetic mosaic screen reveals ecdysone-responsive genes regulating *Drosophila* oogenesis

Elizabeth T. Ables, Grace Hwang, Danielle S. Finger, Taylor D. Hinnant, and Daniela Drummond-Barbosa

Table S1. Complete list of screened ecdysone-responsive candidate genes, including gene identifiers, gene ontology enrichment analyses, and comparisons to other screens. Please see accompanying Microsoft Excel spreadsheet.

Table S2. Quantification of GSC loss in screened mutants.

**Gene Symbol**	**Gene Name**	**DGRC[1] number**	**FRT arm**	**% germaria showing a GSC loss event[2]**	**% GSC clones recovered[3]**	***n*[4]**
control		n.a.	40A	5.3	32.7	55
control		n.a.	82B	0	34.6	52
Hrb27C	*Heterogeneous nuclear ribonucleoprotein at 27C*	111072	40A	63.2**	10.9**	64
vkg	*Viking*	111122	40A	66.7**	3.4**	59
Acer	*Angiotensin-converting enzyme-related*	111221	40A	50.0**	11.5**	52
Trn-SR	*Transportin-Serine/Arginine rich*	111581	40A	100**	0**	54
CG12050	*CG12050*	114378	40A	69.2**	3.4**	119
MESR3	*Misexpression suppressor of ras 3*	114445	40A	60.0**	3.5**	57
Tpr2	*Tetratricopeptide repeat protein 2*	111623	40A	44.4*	9.3**	54
VhaSFD	*Vacuolar H^+-ATPase SFD subunit*	111707	40A	44.4*	9.3**	54

Dph5	*Diphthamide methyltransferase*	111049	82B	28.6*	13.5**	74
Hr39	*Hormone receptor-like in 39*	114391	40A	35.7*	16.4*	55
CycE	*Cyclin E*	111513	40A	50.0**	20.0	55
trx	*Trithorax*	111414	82B	40.0**	26.8	56
Droj2	*DnaJ-like-2*	111410	82B	27.8*	28.3	53
Kr-h1	*Kruppel homolog 1*	111516	40A	14.3	11.8**	51
CG9305, CG6565	*CG9305, CG6565*	111710	40A	0	9.4**	53
Df31	*Decondensation factor 31*	114345	40A	0	3.4**	59
crp	*cropped*	111622	40A	0	1.6**	63
crp	*cropped*	111066	40A	23.5	25.5	51
crol	*crooked legs*	111079	40A	12.0	43.1	51
x16	*x16*	111121	40A	18.8	23.6	55
CG9302, βCOP	*CG9302, Coat Protein (coatomer) β*	111124	40A	0	21.6	51
CG9302, βCOP	*CG9302, Coat Protein (coatomer) β*	111724	40A	11.1	32.0	50
Cg25c	*Collagen type IV*	111127	40A	12.5	42.0	50
Hop	*Hsp70/Hsp90 organizing protein homolog*	111130	40A	0	17.2	58
vri	*vrille*	111187	40A	20.0	6.9	58
hoip	*hoi-polloi*	111208	40A	10.5	32.7	52

FKBP59	*FK506-binding protein FKBP59*	111255	40A	50.0	1.9	53
kis	*kismet*	111304	40A	30.0	12.7	55
ebi	*ebi*	111356	40A	14.3	21.4	56
dbe	*dribble*	111429	40A	27.3	28.6	56
lace	*lace*	111432	40A	5.6	33.3	51
CG17259	*CG17259*	111502	40A	9.5	37.3	51
aop	*anterior open*	111615	40A	0	42.0	50
RapGAP1, Pen	*Rap GTPase activating protein 1, Pendulin*	111704	40A	23.5	25.5	51
nesd	*nessun dorma*	111706	40A	11.8	28.3	53
FASN1	*Fatty acid synthase 1*	111713	40A	5.9	30.8	52
CG11377	*CG11377*	111725	40A	23.1	18.9	53
brat	*brain tumor*	114346	40A	13.3	24.5	53
Dref	*DNA replication-related element factor*	114408	40A	12.5	26.9	52
dsf	*dissatisfaction*	114516	40A	14.3	36.0	50
CG10341	*CG10341*	114554	40A	11.1	45.3	53
CG9253	*CG9253*	114566	40A	13.6	37.3	51
kuz	*kuzbanian*	114578	40A	4.8	40.0	50
kra	*krasavietz*	111026	82B	3.3	50.9	57
Atu	*Another transcription unit*	111027	82B	6.9	52.9	51
CG11722, mtTFB2	*CG11722, mitochondrial*	111033	82B	9.4	50.0	58

	transcription factor B2					
mod(mdg4)	*modifier of mdg4*	111048	82B	0	50.0	50
OstStt3	*Oligosaccharyl transferase 3*	111054	82B	17.2	46.2	52
cindr	*CIN85 and CD2AP orthologue*	111060	82B	0	54.7	53
Alh	*Alhambra*	111406	82B	0	52.0	50
14-3-3ε	*14-3-3ε*	111416	82B	17.7	24.6	57
Atpα	*Na pump α subunit*	111419	82B	13.0	39.2	51
pnt	*pointed*	111552	82B	10.3	45.6	57
γCOP	*Coat Protein (coatomer) γ*	111595	82B	5.9	57.1	56
CG7800	*CG7800*	111657	82B	3.0	59.3	54
CtBP	*C-terminal Binding Protein*	111616	82B	11.1	40.0	60

[1]Reference number for stocks obtained from the *Drosophila* Genetic Resource Center, Kyoto, Japan.
[2]GSC loss scored as the percentage of germline mosaic germaria harboring at least one GFP-negative daughter cyst, but lacking a corresponding GFP-negative mother GSC.
[3]GSC loss scored as the percentage of total germaria harboring a GFP-negative GSC.
[4]Number of germaria scored.
*p<0.05, **p<0.01, as compared to mock control (Chi-square test).

Table S3. Quantification of FSC loss in screened mutants.

Gene Symbol	Gene Name	DGRC[1] number	FRT arm	% germaria showing a FSC loss event[2]	% FSC clones recovered[3]	*n*[4]
Control		n.a.	40A	5.6	30.9	55
Control		n.a.	82B	2.6	71.2	52
dbe	*dribble*	111429	40A	66.7**	1.8**	56
CG12050	*CG12050*	114378	40A	90.0**	0.8**	119
MESR3	*Misexpression suppressor of ras 3*	114445	40A	50.0**	7.0**	57
pnt	*pointed*	111552	82B	29.6**	36.8**	57
γCOP	*Coat Protein (coatomer) γ*	111595	82B	26.9**	33.9**	56
CtBP	*C-terminal Binding Protein*	111616	82B	31.6**	23.3**	60
Df31	*Decondensation factor 31*	114345	40A	50.0*	1.7**	59
mod(mdg4)	*modifier of mdg4*	111048	82B	21.0*	50.0*	50
vri	*Vrille*	111187	40A	33.3*	17.2	58
Acer	*Angiotensin-converting enzyme-related*	111221	40A	33.3*	15.4	52
Hrb27C	*Heterogeneous nuclear ribonucleoprotein at 27C*	111072	40A	33.3	3.1**	64
crol	*crooked legs*	111079	40A	0	62.6**	51

CycE	*Cyclin E*	111513	40A	16.7	9.1**	55
Trn-SR	*Transportin-Serine/Arginine rich*	111581	40A	28.6	9.3**	54
crp	*cropped*	111622	40A	33.3	6.4**	63
kra	*krasavietz*	111026	82B	5.9	28.1**	57
Dph5	*Diphthamide methyltransferase*	111049	82B	0	25.7**	74
trx	*trithorax*	111414	82B	8.7	37.5**	56
CG9305, CG6565	*CG9305, CG6565*	111710	40A	0	13.2*	53
Droj2	*DnaJ-like-2*	111410	82B	7.1	49.1*	53
14-3-3ε	*14-3-3ε*	111416	82B	0	52.6*	57
dsf	*dissatisfaction*	114516	40A	3.7	52.0*	50
crp	*cropped*	111066	40A	15.4	21.6	51
x16	*x16*	111121	40A	11.1	29.1	55
vkg	*viking*	111122	40A	0	25.4	59
CG9302, βCOP	*CG9302, Coat Protein (coatomer) β*	111124	40A	14.3	23.5	51
Cg25c	*Collagen type IV*	111127	40A	0	24.0	50
Hop	*Hsp70/Hsp90 organizing protein homolog*	111130	40A	6.3	25.9	58
hoip	*hoi-polloi*	111208	40A	17.7	26.9	52
kis	*kismet*	111304	40A	7.4	45.5	55
ebi	*ebi*	111356	40A	15.4	19.6	56
lace	*lace*	111432	40A	13.0	39.2	51

CG17259	*CG17259*	111502	40A	6.3	29.4	51
Kr-h1	*Kruppel homolog 1*	111516	40A	5.3	35.3	51
Tpr2	*Tetratricopeptide repeat protein 2*	111623	40A	14.3	33.3	54
RapGAP1, Pen	*Rap GTPase activating protein 1, Pendulin*	111704	40A	4.2	45.1	51
nesd	*nessun dorma*	111706	40A	13.0	37.7	53
VhaSFD	*Vacuolar H^+-ATPase SFD subunit*	111707	40A	18.2	16.7	54
FASN1	*Fatty acid synthase 1*	111713	40A	5.3	34.6	52
CG9302, βCOP	*CG9302, Coat Protein (coatomer) β*	111724	40A	4.2	46.0	50
brat	*brain tumor*	114346	40A	4.8	37.7	53
Hr39	*Hormone receptor-like in 39*	114391	40A	18.2	32.7	55
Dref	*DNA replication-related element factor*	114408	40A	18.2	34.6	52
CG10341	*CG10341*	114554	40A	0	17.0	53
CG9253	*CG9253*	114566	40A	22.7	33.3	51
kuz	*kuzbanian*	114578	40A	15.8	32.0	50
Atu	*Another transcription unit*	111027	82B	5.6	66.7	51
CG11722, mtTFB2	*CG11722, mitochondrial transcription factor B2*	111033	82B	2.7	62.1	58

OstStt3	*Oligosaccharyl transferase 3*	111054	82B	0	61.5	52
cindr	*CIN85 and CD2AP orthologue*	111060	82B	5.7	62.3	53
Alh	*Alhambra*	111406	82B	4.9	78.0	50
Atpα	*Na pump α subunit*	111419	82B	9.7	54.9	51
CG7800	*CG7800*	111657	82B	8.3	61.1	54

[1]Reference number for stocks obtained from the *Drosophila* Genetic Resource Center, Kyoto, Japan.
[2]FSC loss scored as the percentage of somatic mosaic germaria harboring at least one GFP-negative daughter follicle cell, but lacking a corresponding GFP-negative mother FSC.
[3]FSC loss scored as the percentage of total germaria harboring a GFP-negative FSC.
[4]Number of germaria scored.
**p*<0.05, ***p*<0.01, as compared to mock control (Chi-square test).

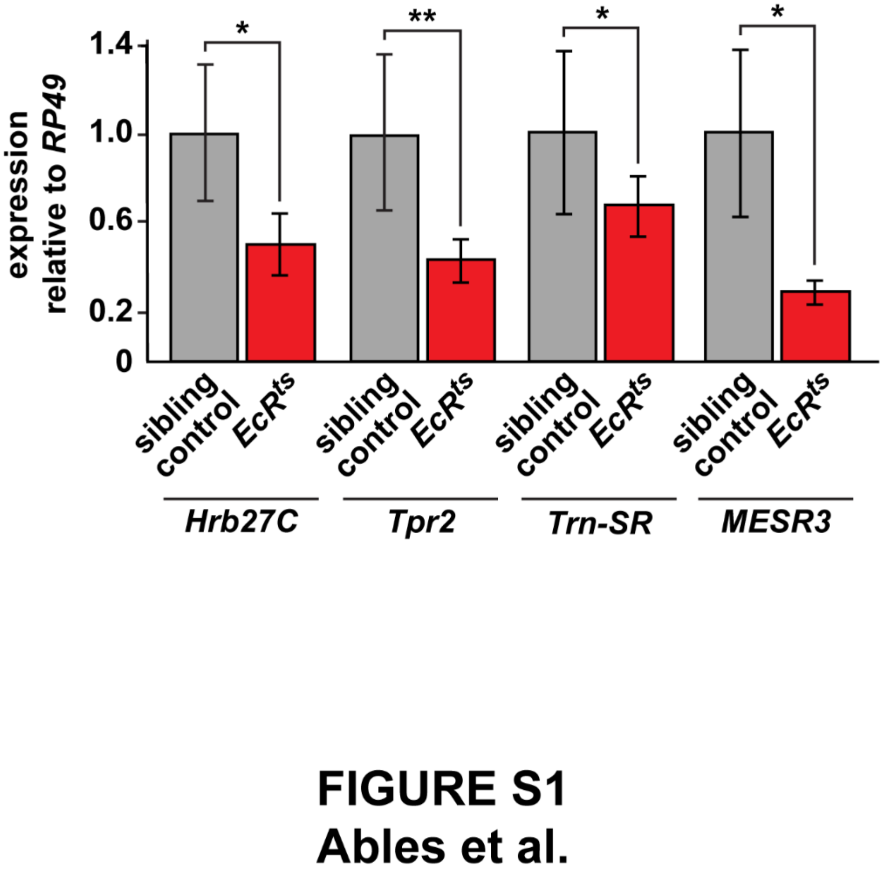

Figure S1. Ovarian expression of putative ecdysone response genes requires optimal ecdysone signaling. Relative expression of selected candidate ecdysone response genes (*Hrb27C*, *Tpr2*, *Trn-SR*, and *MESR3)* in sibling control and *EcR*^{ts} mutant ovaries. Bars indicate average relative quantitative reverse-transcriptase PCR ΔΔCq ratios from three biological replicates, normalized to reference gene *rp49* expression and to sibling control biological controls. Error bars, mean ± SEM. *$p < 0.05$, **$p < 0.0001$; Student's two-tailed T-test.

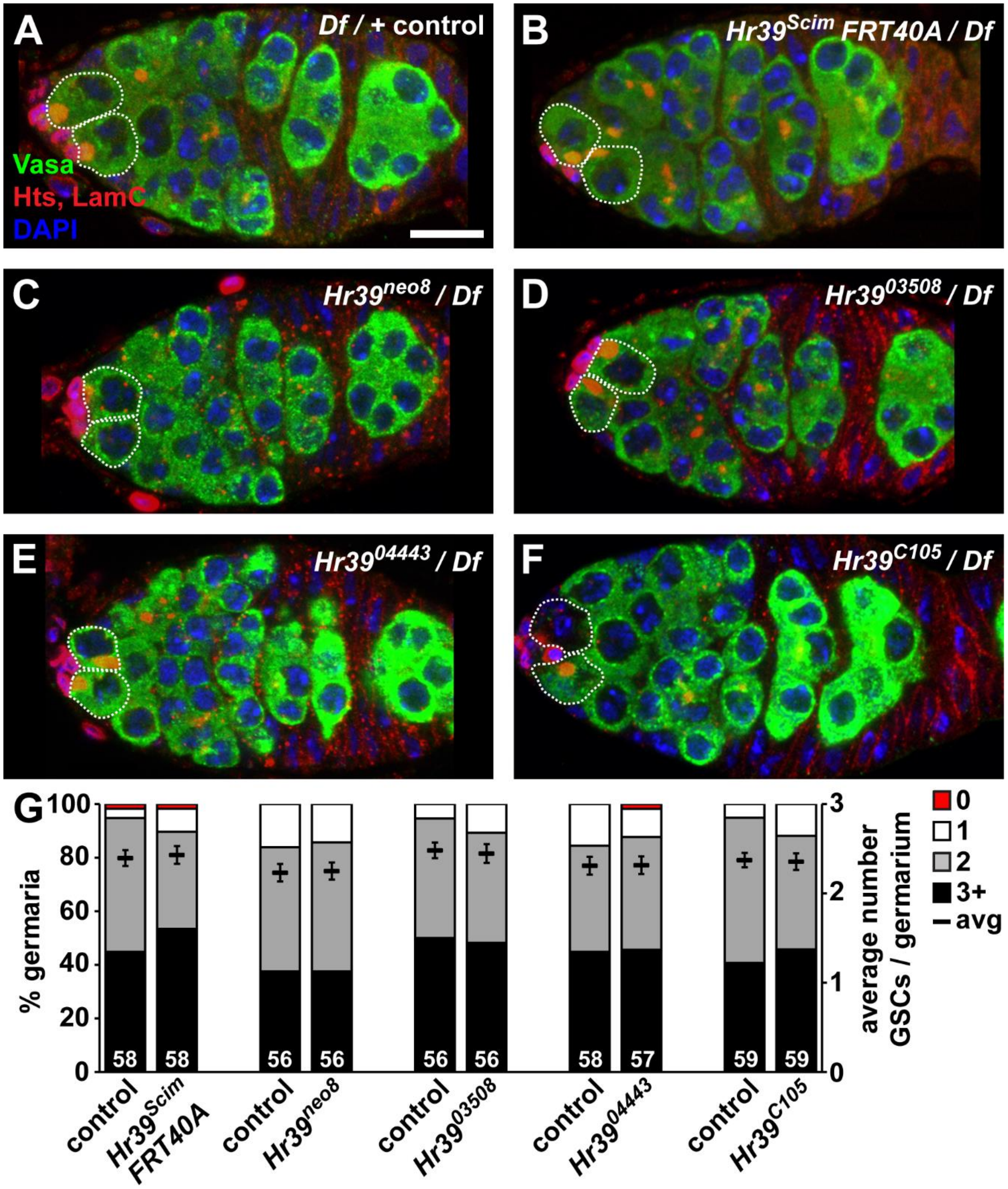

FIGURE S2
Ables et al.

Figure S2. *Hr39* is not required for GSC maintenance. (A-E) Heterozygous sibling control (carrying a balancer, +) (A) or *Hr39* hemizygous mutant (B-E) germaria labeled with anti-Vasa (green; germ cells), anti-Hts (red; fusomes and follicle cell membranes), and anti-LamC (red; nuclear envelope of cap cells). Dotted lines demarcate GSCs. Scale bar, 10 µm. (C) Frequencies of germaria containing 0 (red), 1 (white), 2 (gray), or 3 or more (black) GSCs per germarium (left y-axis) and average number of GSCs per germarium (right y-axis) in heterozygous sibling control and *Hr39* hemizygous mutant females at 10 days after eclosion. The number of germaria analyzed is shown inside bars. Error bars, mean ± SEM. No significant differences in average number of GSCs per germarium were observed; Student's two-tailed T-test.

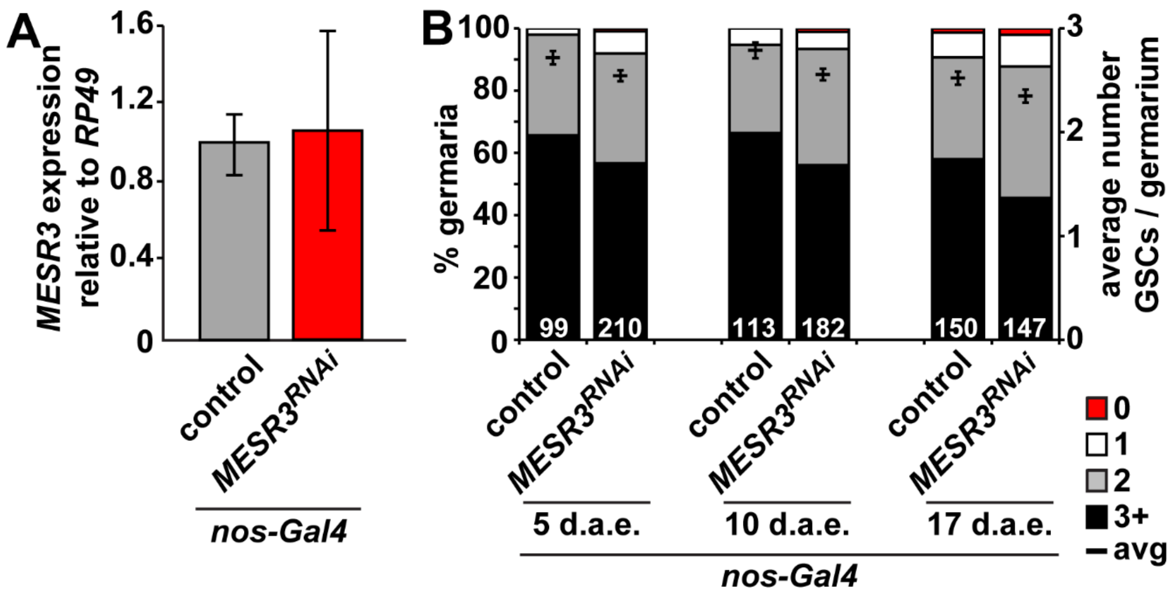

FIGURE S3
Ables et al.

Figure S3. Germline-specific knockdown of *MESR3* does not result in decreased *MESR3* transcript levels in whole ovaries or significant reduction in GSC number. (A) Relative expression of *MESR3* transcripts in *nos-Gal4* control and *nos-Gal4*>*UAS-MESR3*^{RNAi} whole ovaries. Bars indicate average relative quantitative reverse-transcriptase PCR ΔΔCq ratios from three biological replicates, normalized to reference gene *rp49* expression and to *nos-Gal4* biological controls. (B) Frequencies of germaria containing 0 (red), 1 (white), 2 (gray), or 3 or more (black) GSCs per germarium (left y-axis) and average number of GSCs per germarium (right y-axis) in *nos-Gal4* control or *nos-Gal4*>*UAS-MESR3*^{RNAi} knockdown females at 5, 10, and 17 days after eclosion (d. a. e.). The number of germaria analyzed is shown inside bars. No

significant differences in average number of GSCs per germarium were observed. Error bars, mean ± SEM.

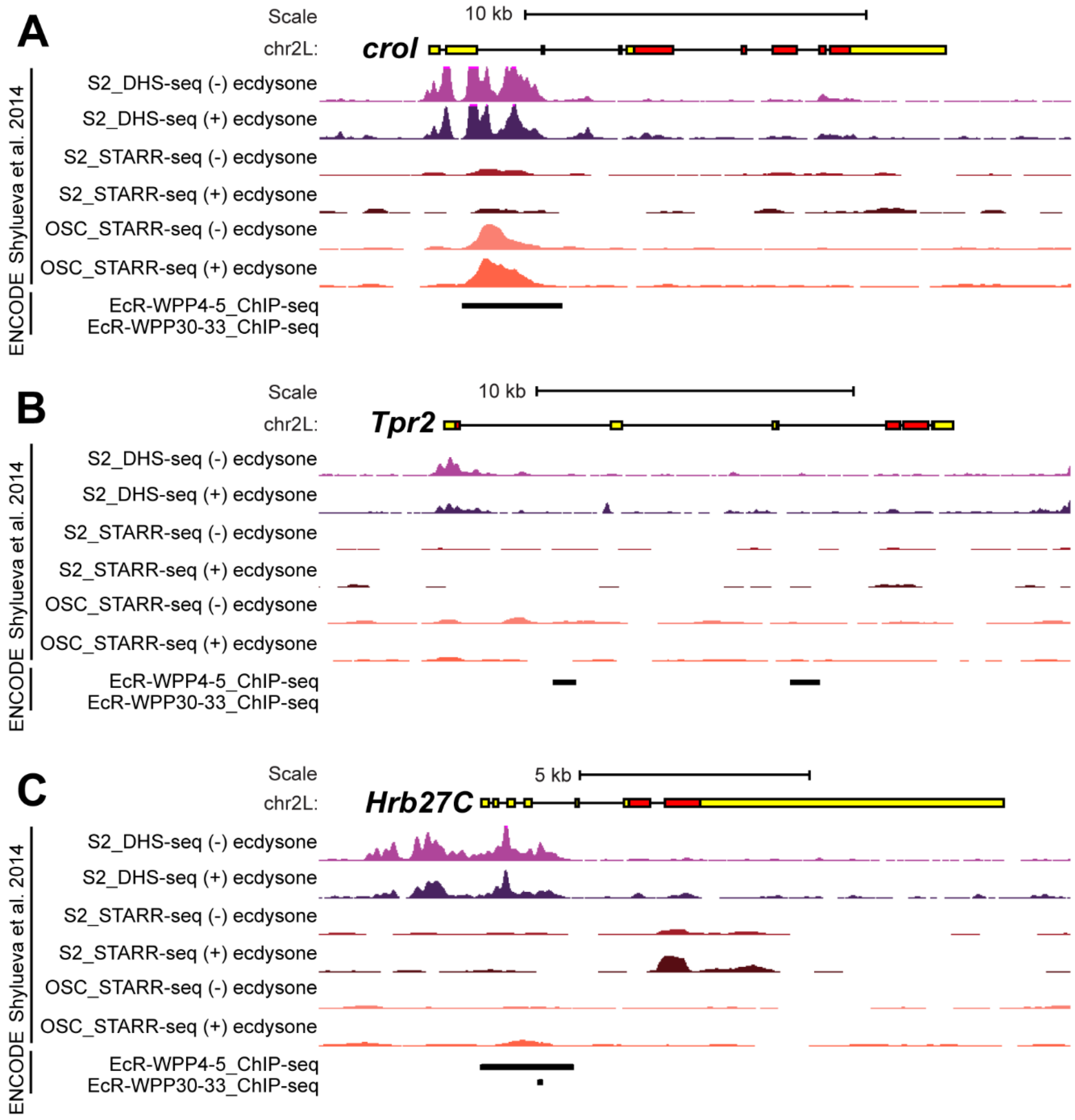

FIGURE S4
Ables et al.

Figure S4. Ecdysone-responsive genes with ovarian phenotypes may be direct targets of EcR. (A-C) University of California at Santa Cruz (UCSC) Genome Browser (https://genome.ucsc.edu/) screenshots of DHS-seq in S2 cells, STARR-seq in S2 and ovarian

somatic (OSC) cells, and EcR ChIP-seq in white pre-pupae (WPP) 4-5 hours after pupariation or 30-33 hours after pupariation for the *crol* (A), *Tpr2* (B), and *Hrb27C* (C) gene loci (representative isoforms are shown). DHS-seq identifies DNaseI-accessible, nucleosome-free regions of DNA (a common property of of active enhancers). STARR-seq places candidate enhancers of variable lengths from genomic DNA downstream of a minimal promoter in a bacterial reporter gene; active enhancers thus up-regulate their own expression. ChIP-seq identifies regions of DNA bound *in vivo* by a transcription factor of interest. Scale bar indicates sequence length. Sequencing data compiled from publically available resources (ROY *et al.* 2010; NEGRE *et al.* 2011; SHLYUEVA *et al.* 2014b; SLATTERY *et al.* 2014).

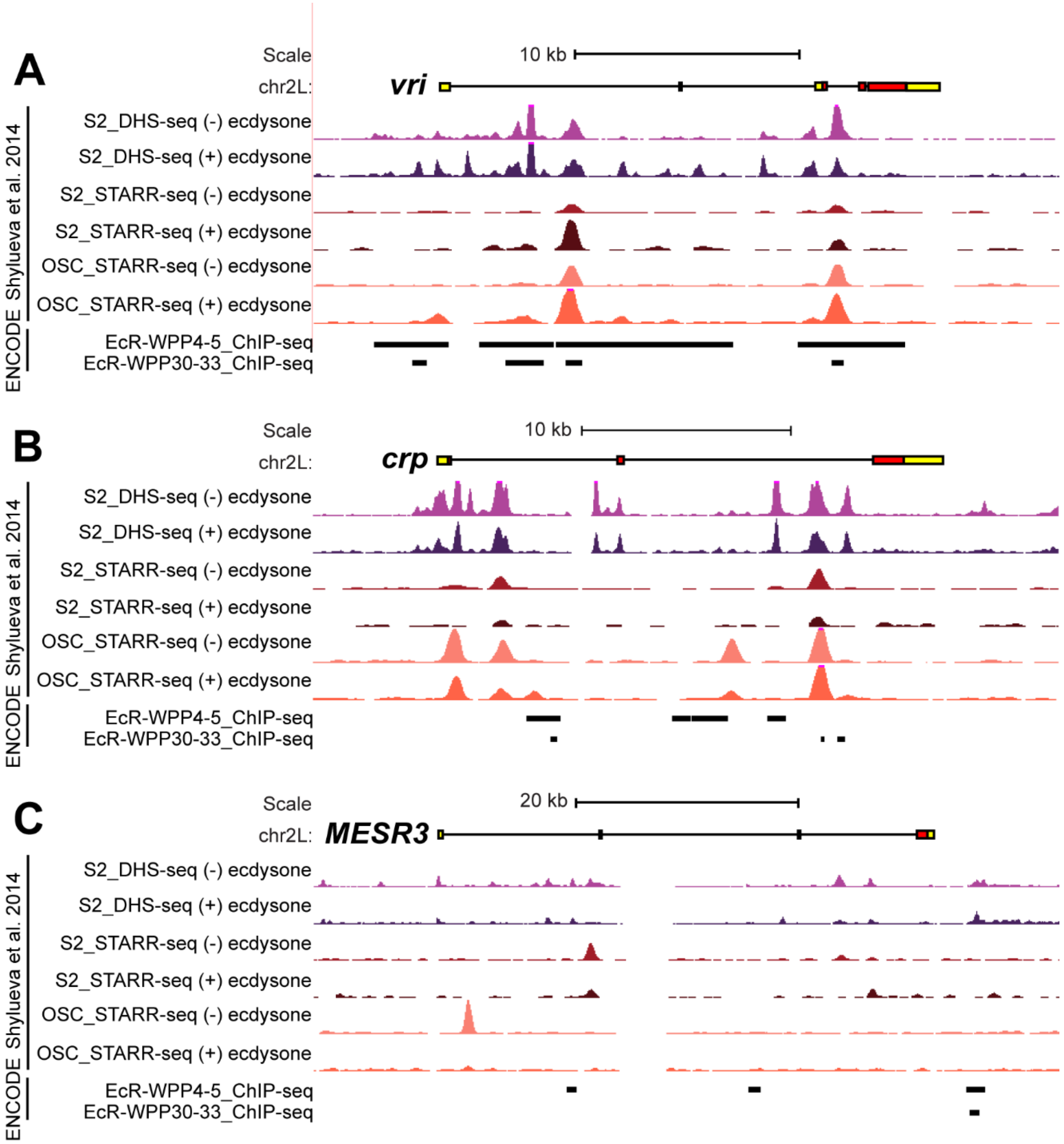

FIGURE S5
Ables et al.

Figure S5. Ecdysone-responsive genes with ovarian phenotypes may be direct targets of EcR. (A-C) University of California at Santa Cruz (UCSC) Genome Browser (https://genome.ucsc.edu/) screenshots of DHS-seq in S2 cells, STARR-seq in S2 and ovarian

somatic (OSC) cells, and EcR ChIP-seq in white pre-pupae (WPP) 4-5 hours after pupariation or 30-33 hours after pupariation for the *vri* (A), *crp* (B), and *MESR3* (C) gene loci (representative isoforms are shown). Scale bar indicates sequence length. Sequencing data compiled from publically available resources (ROY *et al.* 2010; NEGRE *et al.* 2011; SHLYUEVA *et al.* 2014b; SLATTERY *et al.* 2014).
