# Supplementary material for: A Genetic Mosaic Screen Reveals Ecdysone-Responsive Genes Regulating Drosophila Oogenesis
Source: G3 (Bethesda). 2016 May 24;6(8):2629–42. doi: 10.1534/g3.116.028951 (PMC4978916; doi:10.1534/g3.116.028951)
Supplement: Supplemental Material [file supp_g3.116.028951_TableS2.docx]

**Table S2. Quantification of GSC loss in screened mutants.**

| **Gene Symbol** | **Gene Name** | **DGRC^1^ number** | **FRT arm** | **% germaria showing a GSC loss event^2^** | **% GSC clones recovered^3^** | ***n*^4^** |
| --- | --- | --- | --- | --- | --- | --- |
| control |  | n.a. | 40A | 5.3 | 32.7 | 55 |
| control |  | n.a. | 82B | 0 | 34.6 | 52 |
| *Hrb27C* | *Heterogeneous nuclear ribonucleoprotein at 27C* | 111072 | 40A | 63.2** | 10.9** | 64 |
| *vkg* | *Viking* | 111122 | 40A | 66.7** | 3.4** | 59 |
| *Acer* | *Angiotensin-converting enzyme-related* | 111221 | 40A | 50.0** | 11.5** | 52 |
| *Trn-SR* | *Transportin-Serine/Arginine rich* | 111581 | 40A | 100** | 0** | 54 |
| *CG12050* | *CG12050* | 114378 | 40A | 69.2** | 3.4** | 119 |
| *MESR3* | *Misexpression suppressor of ras 3* | 114445 | 40A | 60.0** | 3.5** | 57 |
| *Tpr2* | *Tetratricopeptide repeat protein 2* | 111623 | 40A | 44.4* | 9.3** | 54 |
| *VhaSFD* | *Vacuolar H^+^-ATPase SFD subunit* | 111707 | 40A | 44.4* | 9.3** | 54 |
| *Dph5* | *Diphthamide methyltransferase* | 111049 | 82B | 28.6* | 13.5** | 74 |
| *Hr39* | *Hormone receptor-like in 39* | 114391 | 40A | 35.7* | 16.4* | 55 |
| *CycE* | *Cyclin E* | 111513 | 40A | 50.0** | 20.0 | 55 |
| *trx* | *Trithorax* | 111414 | 82B | 40.0** | 26.8 | 56 |
| *Droj2* | *DnaJ-like-2* | 111410 | 82B | 27.8* | 28.3 | 53 |
| *Kr-h1* | *Kruppel homolog 1* | 111516 | 40A | 14.3 | 11.8** | 51 |
| *CG9305, CG6565* | *CG9305, CG6565* | 111710 | 40A | 0 | 9.4** | 53 |
| *Df31* | *Decondensation factor 31* | 114345 | 40A | 0 | 3.4** | 59 |
| *crp* | *cropped* | 111622 | 40A | 0 | 1.6** | 63 |
| *crp* | *cropped* | 111066 | 40A | 23.5 | 25.5 | 51 |
| *crol* | *crooked legs* | 111079 | 40A | 12.0 | 43.1 | 51 |
| *x16* | *x16* | 111121 | 40A | 18.8 | 23.6 | 55 |
| *CG9302, βCOP* | *CG9302, Coat Protein (coatomer) β* | 111124 | 40A | 0 | 21.6 | 51 |
| *CG9302, βCOP* | *CG9302, Coat Protein (coatomer) β* | 111724 | 40A | 11.1 | 32.0 | 50 |
| *Cg25c* | *Collagen type IV* | 111127 | 40A | 12.5 | 42.0 | 50 |
| *Hop* | *Hsp70/Hsp90 organizing protein homolog* | 111130 | 40A | 0 | 17.2 | 58 |
| *vri* | *vrille* | 111187 | 40A | 20.0 | 6.9 | 58 |
| *hoip* | *hoi-polloi* | 111208 | 40A | 10.5 | 32.7 | 52 |
| *FKBP59* | *FK506-binding protein FKBP59* | 111255 | 40A | 50.0 | 1.9 | 53 |
| *kis* | *kismet* | 111304 | 40A | 30.0 | 12.7 | 55 |
| *ebi* | *ebi* | 111356 | 40A | 14.3 | 21.4 | 56 |
| *dbe* | *dribble* | 111429 | 40A | 27.3 | 28.6 | 56 |
| *lace* | *lace* | 111432 | 40A | 5.6 | 33.3 | 51 |
| *CG17259* | *CG17259* | 111502 | 40A | 9.5 | 37.3 | 51 |
| *aop* | *anterior open* | 111615 | 40A | 0 | 42.0 | 50 |
| *RapGAP1, Pen* | *Rap GTPase activating protein 1, Pendulin* | 111704 | 40A | 23.5 | 25.5 | 51 |
| *nesd* | *nessun dorma* | 111706 | 40A | 11.8 | 28.3 | 53 |
| *FASN1* | *Fatty acid synthase 1* | 111713 | 40A | 5.9 | 30.8 | 52 |
| *CG11377* | *CG11377* | 111725 | 40A | 23.1 | 18.9 | 53 |
| *brat* | *brain tumor* | 114346 | 40A | 13.3 | 24.5 | 53 |
| *Dref* | *DNA replication-related element factor* | 114408 | 40A | 12.5 | 26.9 | 52 |
| *dsf* | *dissatisfaction* | 114516 | 40A | 14.3 | 36.0 | 50 |
| *CG10341* | *CG10341* | 114554 | 40A | 11.1 | 45.3 | 53 |
| *CG9253* | *CG9253* | 114566 | 40A | 13.6 | 37.3 | 51 |
| *kuz* | *kuzbanian* | 114578 | 40A | 4.8 | 40.0 | 50 |
| *kra* | *krasavietz* | 111026 | 82B | 3.3 | 50.9 | 57 |
| *Atu* | *Another transcription unit* | 111027 | 82B | 6.9 | 52.9 | 51 |
| *CG11722, mtTFB2* | *CG11722, mitochondrial transcription factor B2* | 111033 | 82B | 9.4 | 50.0 | 58 |
| *mod(mdg4)* | *modifier of mdg4* | 111048 | 82B | 0 | 50.0 | 50 |
| *OstStt3* | *Oligosaccharyl transferase 3* | 111054 | 82B | 17.2 | 46.2 | 52 |
| *cindr* | *CIN85 and CD2AP orthologue* | 111060 | 82B | 0 | 54.7 | 53 |
| *Alh* | *Alhambra* | 111406 | 82B | 0 | 52.0 | 50 |
| *14-3-3ε* | *14-3-3ε* | 111416 | 82B | 17.7 | 24.6 | 57 |
| *Atpα* | *Na pump α subunit* | 111419 | 82B | 13.0 | 39.2 | 51 |
| *pnt* | *pointed* | 111552 | 82B | 10.3 | 45.6 | 57 |
| *γCOP* | *Coat Protein (coatomer) γ* | 111595 | 82B | 5.9 | 57.1 | 56 |
| *CG7800* | *CG7800* | 111657 | 82B | 3.0 | 59.3 | 54 |
| *CtBP* | *C-terminal Binding Protein* | 111616 | 82B | 11.1 | 40.0 | 60 |

^1^Reference number for stocks obtained from the *Drosophila* Genetic Resource Center, Kyoto, Japan.

^2^GSC loss scored as the percentage of germline mosaic germaria harboring at least one GFP-negative daughter cyst, but lacking a corresponding GFP-negative mother GSC.

^3^GSC loss scored as the percentage of total germaria harboring a GFP-negative GSC.

^4^Number of germaria scored.

**p*<0.05, ***p*<0.01, as compared to mock control (Chi-square test).
