# Supplementary material for: A Genetic Mosaic Screen Reveals Ecdysone-Responsive Genes Regulating Drosophila Oogenesis
Source: G3 (Bethesda). 2016 May 24;6(8):2629–42. doi: 10.1534/g3.116.028951 (PMC4978916; doi:10.1534/g3.116.028951)
Supplement: Supplemental Material [file supp_g3.116.028951_TableS3.docx]

**Table S3. Quantification of FSC loss in screened mutants.**

| **Gene Symbol** | **Gene Name** | **DGRC^1^ number** | **FRT arm** | **% germaria showing a FSC loss event^2^** | **% FSC clones recovered^3^** | ***n*^4^** |
| --- | --- | --- | --- | --- | --- | --- |
| Control |  | n.a. | 40A | 5.6 | 30.9 | 55 |
| Control |  | n.a. | 82B | 2.6 | 71.2 | 52 |
| *dbe* | *dribble* | 111429 | 40A | 66.7** | 1.8** | 56 |
| *CG12050* | *CG12050* | 114378 | 40A | 90.0** | 0.8** | 119 |
| *MESR3* | *Misexpression suppressor of ras 3* | 114445 | 40A | 50.0** | 7.0** | 57 |
| *pnt* | *pointed* | 111552 | 82B | 29.6** | 36.8** | 57 |
| *γCOP* | *Coat Protein (coatomer) γ* | 111595 | 82B | 26.9** | 33.9** | 56 |
| *CtBP* | *C-terminal Binding Protein* | 111616 | 82B | 31.6** | 23.3** | 60 |
| *Df31* | *Decondensation factor 31* | 114345 | 40A | 50.0* | 1.7** | 59 |
| *mod(mdg4)* | *modifier of mdg4* | 111048 | 82B | 21.0* | 50.0* | 50 |
| *vri* | *Vrille* | 111187 | 40A | 33.3* | 17.2 | 58 |
| *Acer* | *Angiotensin-converting enzyme-related* | 111221 | 40A | 33.3* | 15.4 | 52 |
| *Hrb27C* | *Heterogeneous nuclear ribonucleoprotein at 27C* | 111072 | 40A | 33.3 | 3.1** | 64 |
| *crol* | *crooked legs* | 111079 | 40A | 0 | 62.6** | 51 |
| *CycE* | *Cyclin E* | 111513 | 40A | 16.7 | 9.1** | 55 |
| *Trn-SR* | *Transportin-Serine/Arginine rich* | 111581 | 40A | 28.6 | 9.3** | 54 |
| *crp* | *cropped* | 111622 | 40A | 33.3 | 6.4** | 63 |
| *kra* | *krasavietz* | 111026 | 82B | 5.9 | 28.1** | 57 |
| *Dph5* | *Diphthamide methyltransferase* | 111049 | 82B | 0 | 25.7** | 74 |
| *trx* | *trithorax* | 111414 | 82B | 8.7 | 37.5** | 56 |
| *CG9305, CG6565* | *CG9305, CG6565* | 111710 | 40A | 0 | 13.2* | 53 |
| *Droj2* | *DnaJ-like-2* | 111410 | 82B | 7.1 | 49.1* | 53 |
| *14-3-3ε* | *14-3-3ε* | 111416 | 82B | 0 | 52.6* | 57 |
| *dsf* | *dissatisfaction* | 114516 | 40A | 3.7 | 52.0* | 50 |
| *crp* | *cropped* | 111066 | 40A | 15.4 | 21.6 | 51 |
| *x16* | *x16* | 111121 | 40A | 11.1 | 29.1 | 55 |
| *vkg* | *viking* | 111122 | 40A | 0 | 25.4 | 59 |
| *CG9302, βCOP* | *CG9302, Coat Protein (coatomer) β* | 111124 | 40A | 14.3 | 23.5 | 51 |
| *Cg25c* | *Collagen type IV* | 111127 | 40A | 0 | 24.0 | 50 |
| *Hop* | *Hsp70/Hsp90 organizing protein homolog* | 111130 | 40A | 6.3 | 25.9 | 58 |
| *hoip* | *hoi-polloi* | 111208 | 40A | 17.7 | 26.9 | 52 |
| *kis* | *kismet* | 111304 | 40A | 7.4 | 45.5 | 55 |
| *ebi* | *ebi* | 111356 | 40A | 15.4 | 19.6 | 56 |
| *lace* | *lace* | 111432 | 40A | 13.0 | 39.2 | 51 |
| *CG17259* | *CG17259* | 111502 | 40A | 6.3 | 29.4 | 51 |
| *Kr-h1* | *Kruppel homolog 1* | 111516 | 40A | 5.3 | 35.3 | 51 |
| *Tpr2* | *Tetratricopeptide repeat protein 2* | 111623 | 40A | 14.3 | 33.3 | 54 |
| *RapGAP1, Pen* | *Rap GTPase activating protein 1, Pendulin* | 111704 | 40A | 4.2 | 45.1 | 51 |
| *nesd* | *nessun dorma* | 111706 | 40A | 13.0 | 37.7 | 53 |
| *VhaSFD* | *Vacuolar H^+^-ATPase SFD subunit* | 111707 | 40A | 18.2 | 16.7 | 54 |
| *FASN1* | *Fatty acid synthase 1* | 111713 | 40A | 5.3 | 34.6 | 52 |
| *CG9302, βCOP* | *CG9302, Coat Protein (coatomer) β* | 111724 | 40A | 4.2 | 46.0 | 50 |
| *brat* | *brain tumor* | 114346 | 40A | 4.8 | 37.7 | 53 |
| *Hr39* | *Hormone receptor-like in 39* | 114391 | 40A | 18.2 | 32.7 | 55 |
| *Dref* | *DNA replication-related element factor* | 114408 | 40A | 18.2 | 34.6 | 52 |
| *CG10341* | *CG10341* | 114554 | 40A | 0 | 17.0 | 53 |
| *CG9253* | *CG9253* | 114566 | 40A | 22.7 | 33.3 | 51 |
| *kuz* | *kuzbanian* | 114578 | 40A | 15.8 | 32.0 | 50 |
| *Atu* | *Another transcription unit* | 111027 | 82B | 5.6 | 66.7 | 51 |
| *CG11722, mtTFB2* | *CG11722, mitochondrial transcription factor B2* | 111033 | 82B | 2.7 | 62.1 | 58 |
| *OstStt3* | *Oligosaccharyl transferase 3* | 111054 | 82B | 0 | 61.5 | 52 |
| *cindr* | *CIN85 and CD2AP orthologue* | 111060 | 82B | 5.7 | 62.3 | 53 |
| *Alh* | *Alhambra* | 111406 | 82B | 4.9 | 78.0 | 50 |
| *Atpα* | *Na pump α subunit* | 111419 | 82B | 9.7 | 54.9 | 51 |
| *CG7800* | *CG7800* | 111657 | 82B | 8.3 | 61.1 | 54 |

^1^Reference number for stocks obtained from the *Drosophila* Genetic Resource Center, Kyoto, Japan.

^2^FSC loss scored as the percentage of somatic mosaic germaria harboring at least one GFP-negative daughter follicle cell, but lacking a corresponding GFP-negative mother FSC.

^3^FSC loss scored as the percentage of total germaria harboring a GFP-negative FSC.

^4^Number of germaria scored.

**p*<0.05, ***p*<0.01, as compared to mock control (Chi-square test).
