# Supplementary figures and images for: A Genetic Mosaic Screen Reveals Ecdysone-Responsive Genes Regulating Drosophila Oogenesis
Source: G3 (Bethesda). 2016 May 24;6(8):2629–42. doi: 10.1534/g3.116.028951 (PMC4978916; doi:10.1534/g3.116.028951)

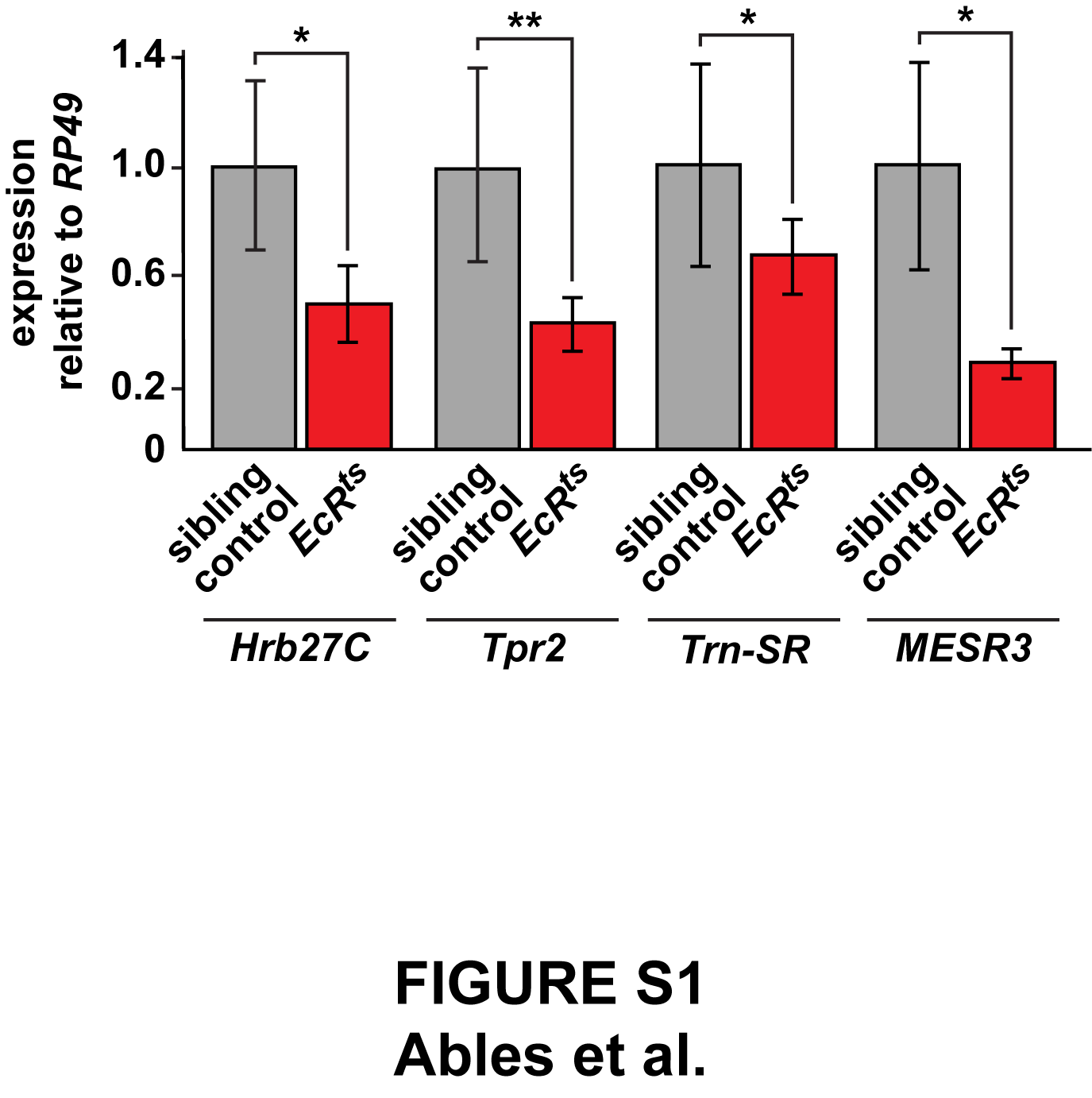

Supplement: Supplemental Material [file supp_g3.116.028951_FigureS1.tif]

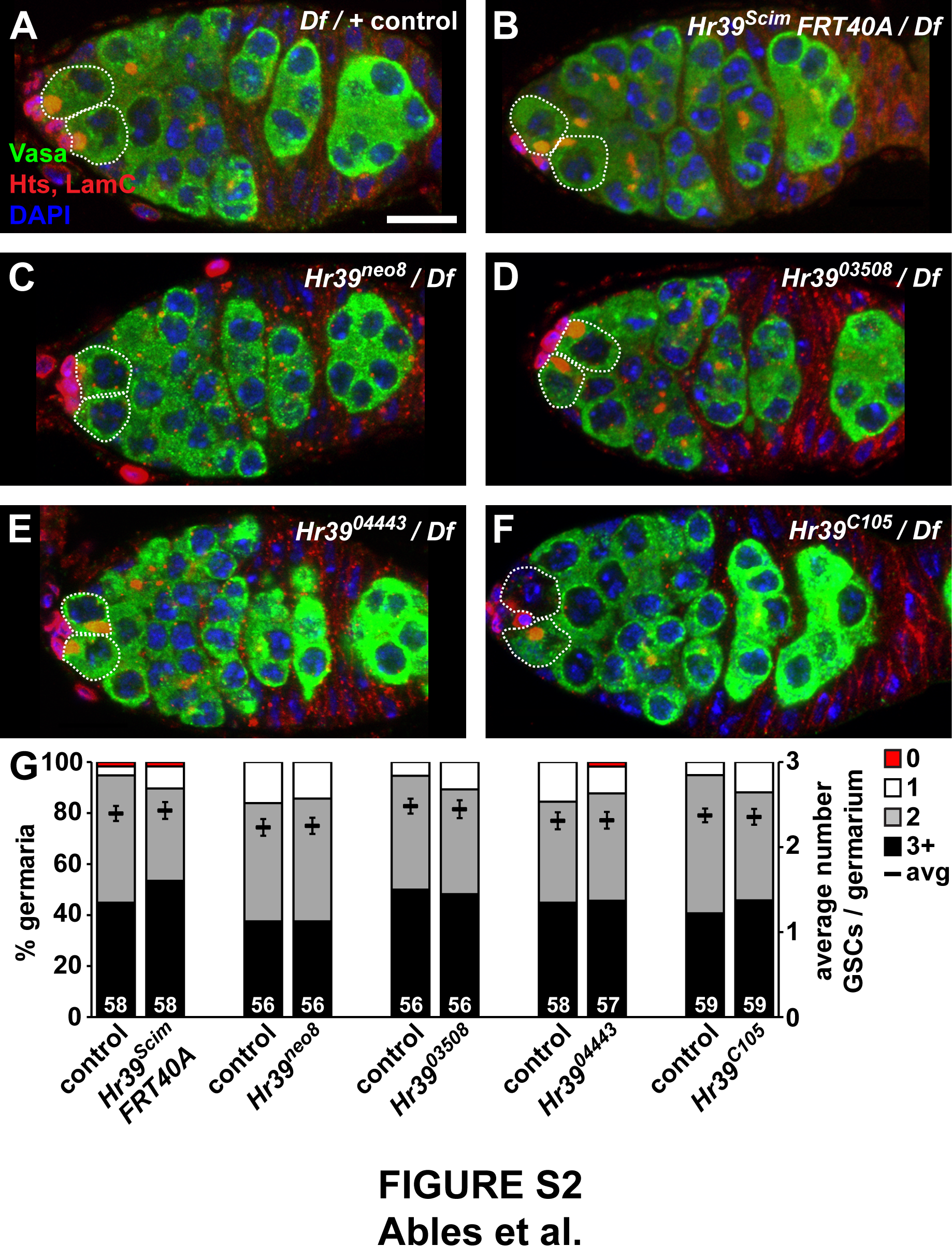

Supplement: Supplemental Material [file supp_g3.116.028951_FigureS2.tif]

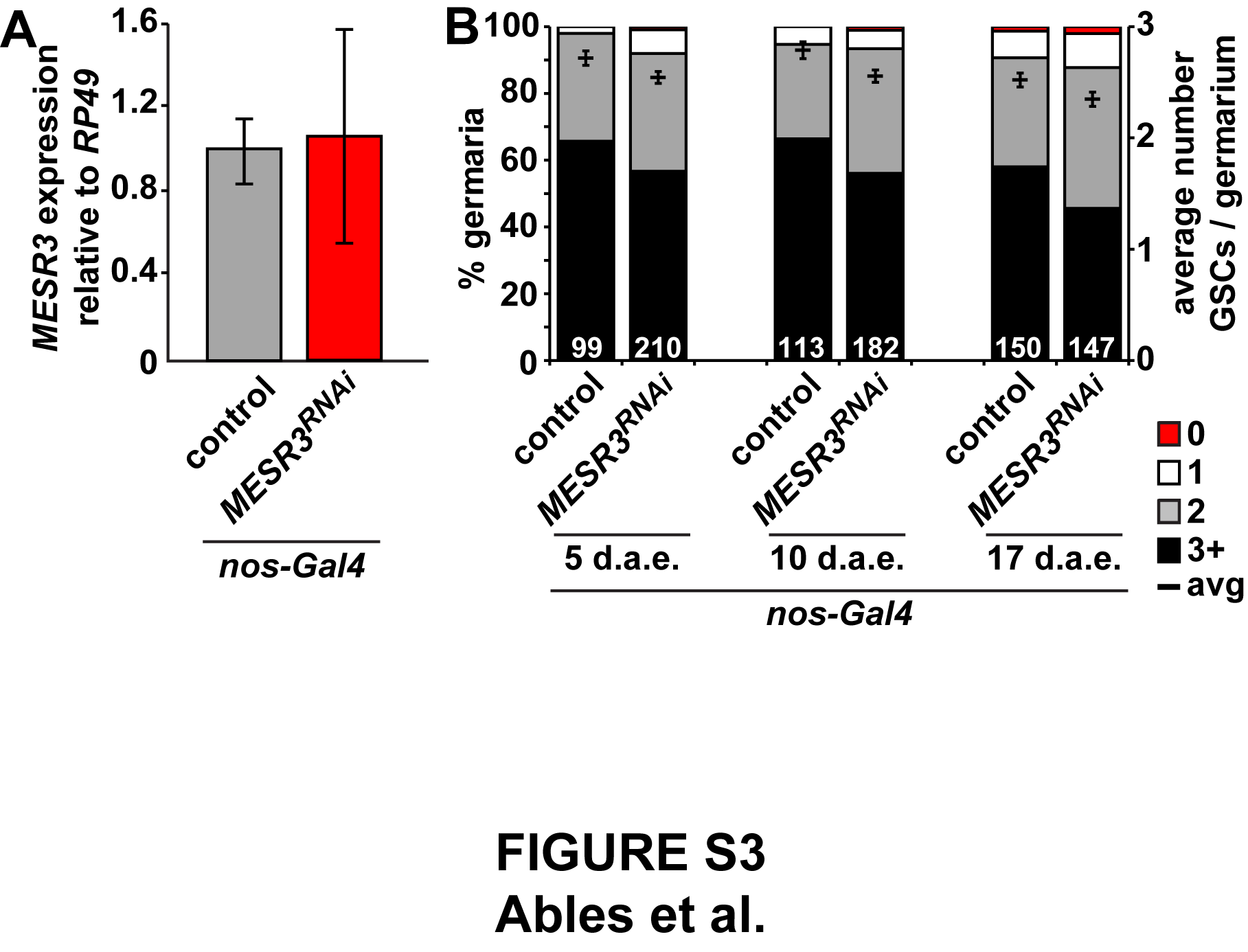

Supplement: Supplemental Material [file supp_g3.116.028951_FigureS3.tif]

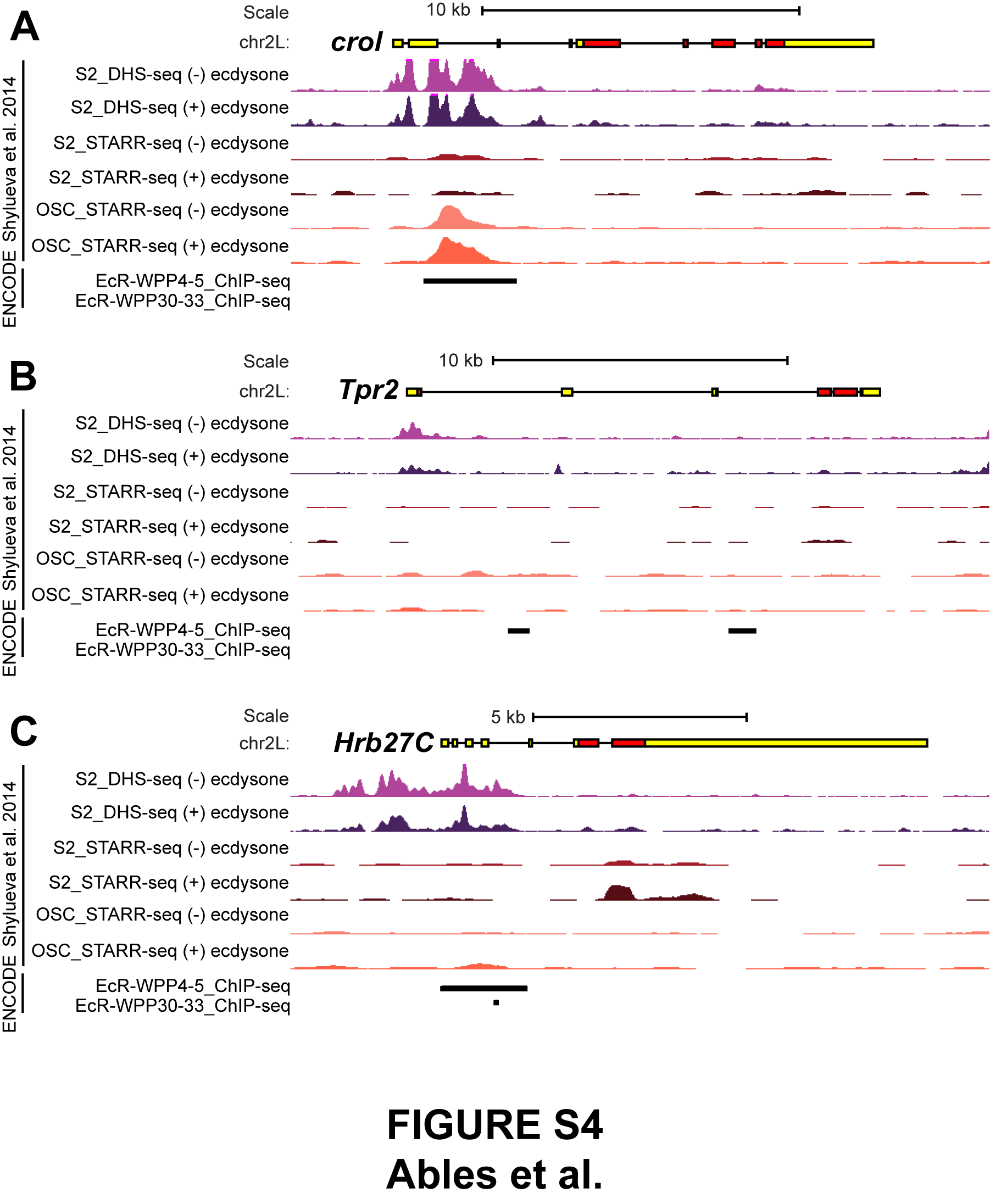

Supplement: Supplemental Material [file supp_g3.116.028951_FigureS4.tif]

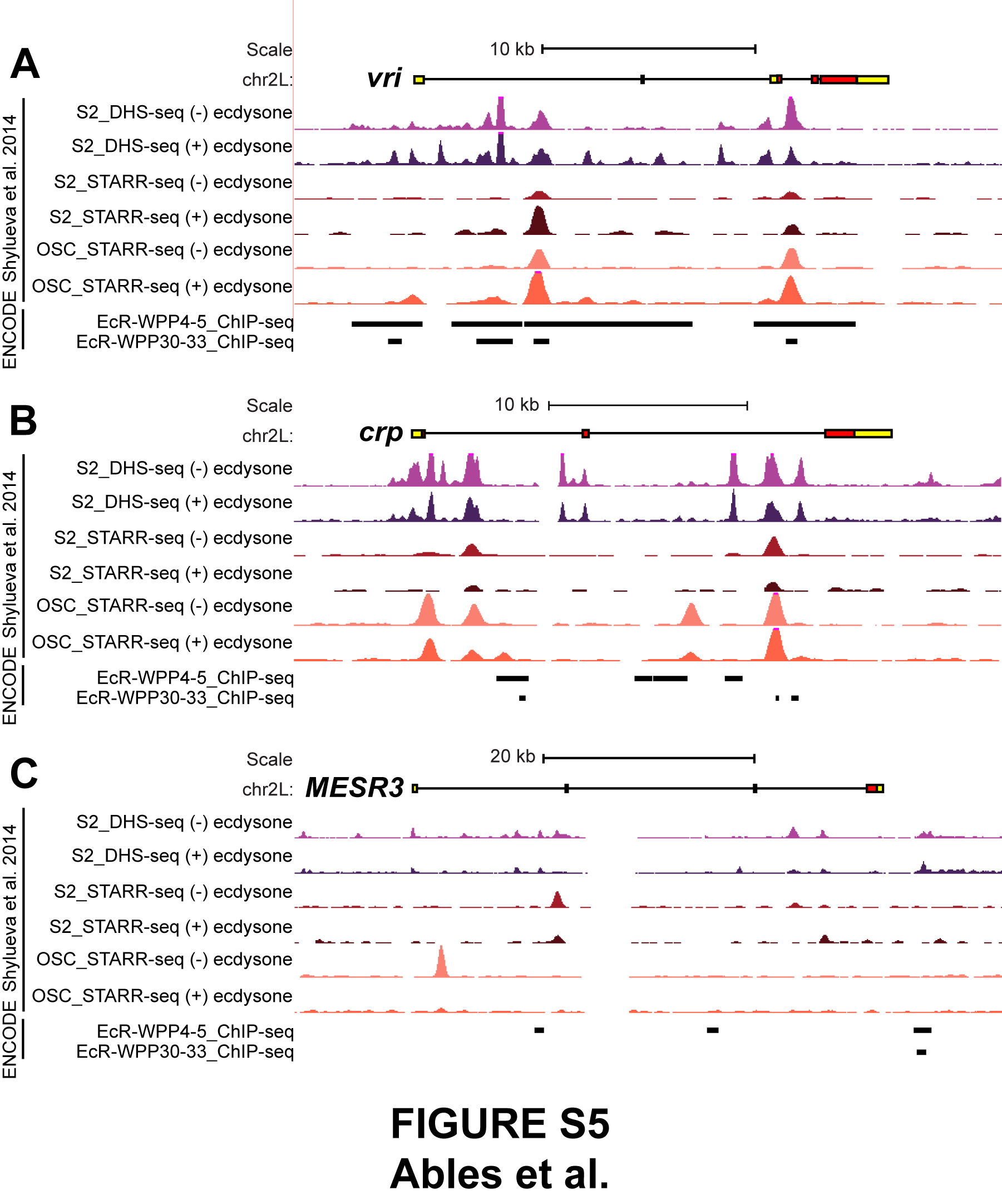

Supplement: Supplemental Material [file supp_g3.116.028951_FigureS5.tif]
